# Probiotic-mediated tumor microenvironment reprogramming with protease-sensitive interleukin-15 and photothermal therapy

**DOI:** 10.1016/j.xcrm.2025.102191

**Published:** 2025-06-17

**Authors:** Huifang Wang, Liuhai Zheng, Chuanbin Yang, Lin Jia, Runhua Zhou, Hongda Liu, Yafang Dong, Xiaolong Xu, Guangwei Shi, Jialu Yang, Yang Li, Haitao Yuan, Jinpeng Cen, Guiming Zhang, Le Yu, Tianqi Guo, Haibo Jiang, Yawei Liu, Xijun Wang, Zhijie Li, Jigang Wang

**Affiliations:** 1 Department of Critical Care Medicine, Guangdong Provincial Clinical Research Center for Geriatrics, Shenzhen Clinical Research Centre for Geriatrics, Shenzhen People’s Hospital (The First Affiliated Hospital, Southern University of Science and Technology, The Second Clinical Medical College, Jinan University), Shenzhen, Guangdong 518020, China; 2 College of Pharmacy, Shenzhen Technology University, Shenzhen, Guangdong 518118, China; 3 State Key Laboratory of Integration and Innovation of Classic Formula and Modern Chinese Medicine, Metabolomics Laboratory, Department of Pharmaceutical Analysis, Heilongjiang University of Chinese Medicine, Heping Road 24, Harbin 150040, China; 4 Department of Neurosurgery & Medical Research Center, Shunde Hospital, Southern Medical University (The First People’s Hospital of Shunde Foshan), Guangzhou, Guangdong 528308, China; 5 The First Clinical Medical College, Southern Medical University, Guangzhou, Guangdong 510515, China; 6 Center for Drug Research and Development, Guangdong Provincial Key Laboratory for Research and Evaluation of Pharmaceutical Preparations, Guangdong Pharmaceutical University, Guangzhou, Guangdong 510006, China; 7 Department of Urology, Nanfang Hospital, Southern Medical University, Guangzhou, Guangdong 510515, China; 8 Guangdong Provincial Key Laboratory of New Drug Screening, Department of Traditional Chinese Medicine and School of Pharmaceutical Sciences, Southern Medical University, Guangzhou 510515, China; 9 Department of Chemistry, The University of Hong Kong, Pok Fu Lam, Hong Kong, China; 10 State Key Laboratory for Quality Ensurance and Sustainable Use of Dao-di Herbs, Artemisinin Research Center, Institute of Chinese Materia Medica, China Academy of Chinese Medical Sciences, Beijing 100700, China; 11 State Key Laboratory of Antiviral Drugs, School of Pharmacy, Henan University, Kaifeng 475004, China

**Keywords:** probiotic, tumor microenvironment, interleukin-15, immunogenic cell death, immunotherapy, immune checkpoint blockade, living therapeutics

## Abstract

T cell inadequacy or exhaustion often causes the failure of immune checkpoint blockade (ICB)-based immunotherapy. Interleukin-15 (IL-15) has been used to prime the tumor microenvironment (TME) to boost the efficiency of immunotherapy. However, its clinical application is hindered by systemic toxicity and low intratumoral concentrations. Here, we engineer the probiotic *Escherichia coli* Nissle 1917 to deliver IL-15 and croconium dye, enabling the TME-responsive release of IL-15 and amplifying the antitumor effect through photothermal therapy. This promotes the recruitment of antigen-presenting cells and T cells and the expansion of T/natural killer cells induced by IL-15. Consequently, it halts the tumor growth and induces systemic memory T cell production. This approach combined with ICBs generates prominent synergistic effects across various immune-hot and immune-cold tumors. This study provides a strategy for targeted delivery of cytokines, demonstrating its high potential for TME reprogramming when combined with immunogenic cell death inducers.

## Highlights

• Engineered EcN with protease-sensitive IL-15 enables TME-responsive IL-15 delivery
• Photothermal therapy amplifies antitumor effects and recruits immune cells
• IL-15/CR + L therapy reprograms the TME and generates systemic memory T cells
• IL-15/CR + L therapy boosts PD-1 blockade efficacy in immune-hot and cold tumors

## Graphical abstract

**Figure:**
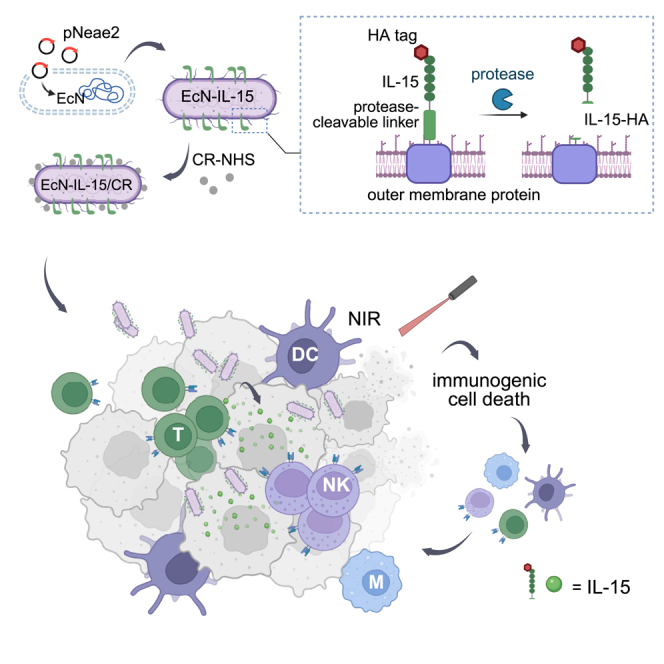

## Teaser

Wang et al. engineered EcN to deliver IL-15 and a photothermal agent, enhancing immunotherapy by reprogramming the tumor microenvironment. This approach boosts immune cell recruitment, generates memory T cells, and demonstrates synergistic effects with immune checkpoint blockade across diverse tumor types.

## Introduction

Cancer immunotherapies like immune checkpoint blockades (ICBs) and adoptive T cell therapy have shown success in some cancers but often fail in “cold” tumors due to poor CD8^+^ T cell priming or dysfunction.^1^ Overcoming limited immune infiltration and functional anergy remains a major challenge. Cytokine-based therapies hold great promise for boosting T cell numbers and functions within the tumor microenvironment (TME) by triggering a broad spectrum of inflammatory responses.^2^^,^^3^ Interleukin-15 (IL-15) stimulates the proliferation of activated and memory CD8^+^ T cells, alongside promoting the proliferation and activation of natural killer (NK) cells.^4^ Recent studies have underscored the importance of IL-15 within the TME in orchestrating optimal antitumor immunity.^5^^,^^6^

However, systemic IL-15 administration is limited by its short half-life and on-target off-tumor toxicity.^7^ This highlights the need to engineer IL-15 for targeted activation of NK or T cells within the TME. Strategies to address these issues include local cytokine injection,^8^ cytokine-producing oncolytic viruses,^9^ adoptive transfer of cytokine-producing cells,^10^ and tumor-targeting cytokines. Numerous studies have explored the co-expression of cytokines (such as IL-2, IL-7, IL-12, IL-15, and IL-21), or the combination of their receptors, to develop cytokine-armored immune cells.^11^ IL-15 alone or together with the IL-15 receptor complex has also been incorporated into many adoptive cell therapies, specifically chimeric antigen receptor T cells and NK cells.^11^ Some of these therapies have showcased enhanced anti-tumor efficacy and cell persistence in clinical investigations.^12^^,^^13^ In addition, the approach of utilizing tumor-targeting cytokines, also known as immunocytokines, has emerged as a strategy aimed at enhancing both safety and efficacy. Coupling cytokines with specific targeting moieties, such as fibronectin-binding IL-15 and IL-2,^14^^,^^15^ tumor vessel-targeting tumor necrosis factor (TNF) alpha and LIGHT,^16^^,^^17^ anti-CD20-RLI,^18^ and collagen-binding IL-12,^19^^,^^20^ enables precise delivery of cytokines to specific cell types within the TME. These approaches can enhance localized cytokine activity while reducing off-target effects. Nonetheless, these strategies still face challenges, including poor tissue penetration in solid tumors and limited efficacy in “cold” tumors. Consequently, there is a need to explore new approaches to address these limitations.

Various bacteria, including *Escherichia coli* (*E. coli*)), *Salmonella*, and *Clostridium*, can preferentially colonize tumors due to hypoxia and poor immune surveillance.^21^^,^^22^ They can be engineered to locally deliver payloads like toxins or immunomodulators that are otherwise toxic or ineffective systemically.^22^ Synthetic biology enables precise control of bacterial behavior in the TME to improve payload efficacy and safety.^23^^,^^24^

Given the limited clinical response to IL-15 monotherapy,^25^^,^^26^^,^^27^ combining IL-15 with other therapies is crucial to enhance its efficacy.^27^^,^^28^^,^^29^^,^^30^ Combining IL-15 with radiotherapy in mice has been found to activate and recruit dendritic cells (DCs) into the tumor, promoting NK and CD8^+^ T cell expansion through IL-15 *trans*-presentation by DCs.^31^ Moreover, a growing number of clinical and preclinical studies show that immunogenic cell death (ICD) can be triggered by chemotherapeutic agents, radiotherapy, and photothermal therapy (PTT), leading to enhanced exposure of damage-associated molecular patterns (DAMPs) and tumor-associated antigens.^32^ DAMPs further drive DC maturation, macrophage polarization, and T cell infiltration, reprogramming the “cold” TME into a “hot” one,^33^ which may improve the therapeutic effects of IL-15. Based on this, we hypothesize a synergistic interaction between ICD-inducing therapies and IL-15.

To address the safety challenges and enhance the therapeutic potential of IL-15-based cancer therapies, the present study leverages the inherent tumor tropism of *E. coli* Nissle 1917 (EcN), a probiotic bacterium, and engineers EcN to display the protease-sensitive cytokine IL-15 on its outer membrane using the bacterial display vector pNeae2.^34^ This approach results in the creation of a biohybrid bacterial system designed to selectively deliver IL-15 to the tumor site, promoting localized immunomodulation. To further amplify the therapeutic efficacy of IL-15, we incorporated the photothermal agent croconium (CR) dye into the system, enabling controlled, mild-temperature photothermal effects. This dual strategy not only directs IL-15 to the TME but also induces localized thermal effects that can further stimulate the immune response. By synergizing IL-15-mediated immunomodulation with PTT, the biohybrid system facilitates immune cell infiltration into the TME, effectively reshaping it into an inflamed, immune-active state (Scheme S1). Moreover, we demonstrate the potential of combining this biohybrid system with anti-programmed cell death protein 1 (PD-1) immunotherapy to alleviate immune suppression in both “hot” and “cold” tumors. This combined strategy holds considerable promise for advancing bacterial-based cancer therapies by integrating cytokine delivery with ICD induction, addressing current challenges related to tumor-specific immune activation and resistance to ICB.

## Results

### Engineering EcN to express TME-cleavable IL-15 cytokine

**Figure 1:**
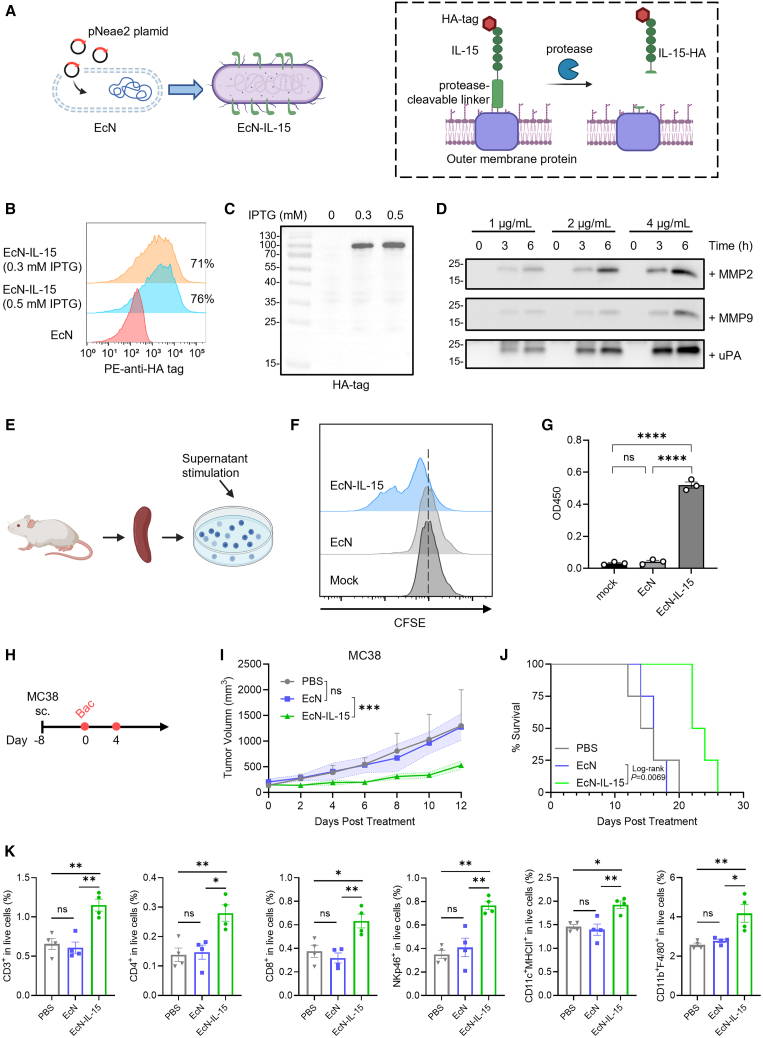
Engineering and characterization of *E. coli* Nissle 1917 to express TME-responsive IL-15 (A) A recombinant plasmid (pNeae2) encoding conditionally released IL-15 protein was introduced into *E. coli* Nissle 1917 (EcN) and expressed on the bacterial outer membrane. (B and C) The expression of HA-tagged IL-15 in EcN-IL-15 was analyzed by flow cytometry and western blot. (D) Western blot analysis of the cleavage of EcN-IL-15 by recombinant proteases. (E–G) The isolated mouse splenocytes were labeled with CFSE and stimulated for 72 h with supernatant from MMP2 cleavage of EcN or EcN-IL-15, followed by flow cytometry analysis and CCK8 assay (*n* = 3). (H–J) EcN-IL-15 strains delayed tumor progression *in vivo* (*n* = 4). (H) Treatment schedule of EcN-IL-15 in subcutaneous MC38 tumor. (I) MC38 tumor growth curves with different treatments (means ± SD). (J) Kaplan-Meier survival curves. (K) EcN-IL-15 reshapes the TME and boosts anti-tumor immune responses. Quantification of CD3^+^ T cells, CD4^+^ T cells, CD8^+^ T cells, NK cells, DCs, and macrophages in MC38 tumor tissues; *n* = 4 biological replicates. Data are presented as means ± SEM unless otherwise specified. *p* values were analyzed by one-way analysis of variance (ANOVA) with Tukey’s multiple comparisons test (G, K), two-way ANOVA with Tukey’s multiple comparisons test (I), or Mantel-Cox log rank test (J). ∗*p* < 0.05, ∗∗*p* < 0.01, ∗∗∗*p* < 0.001, ∗∗∗∗*p* < 0.0001; ns, not significant.

To boost antitumor immunity, we engineered EcN to express IL-15 on its surface using the pNeae2 vector, incorporating a protease-cleavable linker (HPVGLLARVPLSLYSGHPVGLLARVPLSLYSGL SGRSDNH^3^) (Figure 1A). Tumor-enriched proteases (such as matrix metalloproteinase-2 [MMP2], MMP9, and urokinase plasminogen activator [uPA]^3^) can cleave the linker and release hemagglutinin (HA)-tagged IL-15 within the TME. Hereafter, this releasable IL-15 strain is termed EcN-IL-15. A strain with empty pNeae2, without induction, served as the control and was termed EcN.

Flow cytometry analysis and western blot revealed the presence of HA-tagged IL-15 in both bacterial surface and cell pellet upon isopropyl β-D-1-thiogalactopyranoside (IPTG) induction (Figures 1B and 1C). For all subsequent studies, EcN-IL-15 bacteria were pre-induced with IPTG before application. Next, we characterized the proteolytic cleavage of the EcN-IL-15 bacteria by treating them with recombinant proteases (including MMP2, MMP9, and uPA) and visualized free IL-15 in bacterial supernatants by western blotting. Protease co-incubation resulted in the release of free HA-tagged IL-15 into the supernatant in a time- and concentration-dependent manner (Figure 1D). Next, we compared the cleavage of EcN-IL-15 bacteria by tumor or normal tissue homogenate via western blotting. As expected, incubation with MC38 tumor homogenate, which is rich in intratumoral proteases,^3^ resulted in the significant release of HA-tagged IL-15, whereas less cleavage was observed with liver, lung, or kidney homogenates. A similar cleavage pattern was observed with Colon26 tumors (Figure S1). Furthermore, to assess whether the cleaved fragments were bioactive, we stimulated splenocytes with the supernatant from MMP2-cleaved EcN or EcN-IL-15. Carboxyfluorescein succinimidyl ester (CFSE) cell proliferation and CCK8 assays showed that only the EcN-IL-15 supernatant promoted splenocyte proliferation (Figures 1E–1G), suggesting that the cleaved IL-15 fragments are bioactive.

We then examined the *in vivo* efficacy of EcN-IL-15 in an MC38 tumor model (Figure 1H). While EcN alone showed no discernible effect, EcN-IL-15 significantly suppressed tumor growth and improved survival compared to PBS or EcN (Figures 1I, 1J, and S2A). Both EcN-IL-15 and EcN caused slight, transient weight loss, which recovered by day 4 post injection (Figure S2B). In clinical trials, IL-15-related drug administration led to the expansion of circulating NK and T cells, along with hypothermia, liver injury, and the elevation of cytokines contributing to immune-related adverse events.^35^ We then sought to investigate whether EcN-IL-15 bacteria exhibit reduced toxicity compared to IL-15. MC38 tumor-bearing mice received either EcN-IL-15 (4 × 10^7^ colony-forming unit [CFU], two injections) or mouse IL-15 at doses of 5 μg (low dose, L, four injections) or 10 μg (high dose, H, four injections).^36^^,^^37^ Blood samples were collected on day 9, and alanine aminotransferase (ALT) and aspartate aminotransferase (AST) (hepatotoxicity markers), interferon-gamma (IFN-γ), and IL-6 (inflammatory markers) were quantified. IL-15 (H) exhibited a significant antitumor effect compared to PBS and IL-15 (L) groups but was accompanied by a substantial elevation in serum ALT, AST, IFN-γ, and IL-6. In contrast, EcN-IL-15 showed slightly better antitumor efficacy compared to high-dose IL-15 (*p* = 0.06; Figure S3) while significantly reducing systemic toxicity indicated by all tested blood markers. These findings highlight the therapeutic potential of our approach in achieving robust efficacy with an improved safety profile.

To gain a better understanding of the antitumor mechanism of EcN-IL-15, we conducted flow cytometry analyses to assess its effects on immune cell populations both within the tumors and in peripheral tissues. The results revealed that EcN-IL-15 significantly reshapes the TME. Specifically, compared to the PBS and EcN groups, EcN-IL-15 administration notably increased the number of T cells, NK cells, DCs, and macrophages within the tumor sites. In contrast, the EcN group did not result in significant changes in these immune cell populations within the tumors (Figures 1K and S4). Additionally, we observed minimal effects of EcN on immune cell populations in the spleens. While EcN-IL-15 treatment led to a significant increase in DCs in the spleens compared to the PBS group, no significant changes were observed in the numbers of T cells, NK cells, or macrophages (Figures S5A and S5C). In the lymph nodes, EcN-IL-15 treatment resulted in a significant increase in CD3^+^ T cells, CD8^+^ T cells, NK cells, and macrophages compared to the PBS group (Figures S5B and S5C). Overall, our findings suggest that EcN-IL-15 exhibits a pronounced impact on immune cells within the tumor, with significant effects also observed in peripheral lymphoid tissues (lymph nodes).

### Fabrication of photothermal bacteria

**Figure 2:**
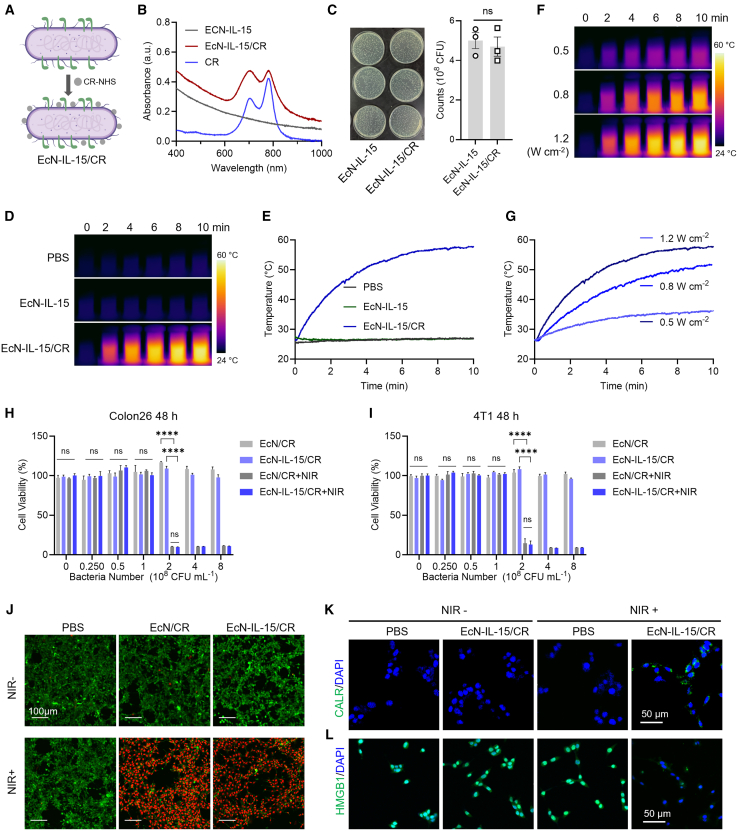
Preparation and characterization of photothermal probiotics EcN-IL-15/CR (A) Schematic diagram of attaching CR-NHS (a photothermal agent) onto the surface of EcN-IL-15. (B) UV-vis absorption spectroscopy showed EcN-IL-15/CR maintained NIR-I absorption similar to CR-NHS. (C) Colony images and quantification of EcN-IL-15 cultured on Luria-Bertani (LB) agar plates before and after CR-NHS modification (*n* = 3). (D–G) Thermal images and temperature variations of CR-modified or unmodified EcN-IL-15 (4 × 10^8^ CFU mL^−1^) under 808 nm laser irradiation (1.2 W cm^−2^) (D and E) and varying power densities (0.5, 0.8, and 1.2 W cm^−2^) for 10 min (F and G). (H and I) Cytotoxicity of EcN-IL-15/CR or EcN/CR on Colon26 and 4T1 cells with or without 808 nm laser irradiation (1.5 W cm^−2^, 10 min) (*n* = 3–4). (J) Live (green)/dead (red) cell fluorescence staining images of Colon26 cells (*n* = 3). Scale bars, 100 μm. (K and L) Evaluation of ICD effect in Colon26 cells treated with EcN-IL-15/CR with or without 808 nm laser irradiation (1.5 W cm^−2^, 10 min) (*n* = 3). Scale bars, 50 μm. Data are presented as means ± SEM. *p* values were analyzed by two-tailed unpaired Student’s t test (C), two-way ANOVA with Tukey’s multiple comparisons test (H and I). ∗∗∗∗*p* < 0.0001; ns, not significant.

We hypothesized that PTT-induced ICD could enhance IL-15’s antitumor efficacy. To integrate photothermal functionality into EcN-IL-15, we conjugated CR dye—a photothermal agent with high conversion efficiency and photostability^38^—to the bacterial surface via CR-N-hydroxysuccinimide ester (NHS), forming EcN-IL-15/CR and control EcN/CR (Figure 2A). The successful conjugation of CR-NHS to bacteria was confirmed by UV-vis-near-infrared (NIR) spectrophotometry, which revealed characteristic CR-NHS absorption peaks (700 and 780 nm) in EcN-IL-15/CR but not in unmodified bacteria (Figure 2B). Moreover, the CR modification did not exert any discernible impact on the survival or growth of the bacteria (Figures 2C and S6A). Importantly, EcN-IL-15/CR bacteria stimulated splenocyte proliferation similarly to EcN-IL-15, suggesting that the CR modification does not impair the immunostimulatory properties of the engineered bacteria (Figures S6B and S6C). Next, the photothermal effects of CR-decorated bacteria were determined. Under 808 nm laser irradiation at 1.2 W cm^−2^, the temperature of EcN-IL-15/CR solution could increase to 57°C within 10 min, while the temperature variations of PBS and EcN-IL-15 did not exceed 3°C under irradiation (Figures 2D and 2E). Further laser irradiation experiments were conducted to scrutinize the photothermal characteristics of EcN-IL-15/CR under varying laser power conditions (0.5, 0.8, and 1.2 W cm^−2^), showing a power-dependent increase in temperature (Figures 2F and 2G). A similar photothermal effect was also observed in EcN/CR (Figure S6D). After ceasing irradiation, the cooling curve was recorded. The time constant (τ) for heat transfer in this system, calculated from the cooling phase data,^38^ was determined to be 202.53 s (Figures S6E and S6F).

Based on the excellent photothermal capability of CR-decorated bacteria, the antitumor effect of the fabricated photothermal bacteria was further investigated *in vitro*. As shown in Figures 2H and 2I, both EcN-IL-15/CR and EcN/CR without irradiation, as well as irradiation alone, exhibited negligible cytotoxicity. The cell viability in the bacteria-treated groups at a high bacterial concentration decreased dramatically to approximately 11% after irradiation. These results were qualitatively validated by live/dead staining of Colon26 cells (Figure 2J). To assess whether photothermal bacteria could induce ICD, classical ICD markers were determined in Colon26 cells using immunofluorescence (IF) staining. As anticipated, compared to the PBS and non-irradiated groups, EcN-IL-15/CR plus irradiation induced an increase in surface calreticulin (CALR) exposure and a decrease in intranuclear high-mobility group box 1 (Figures 2K and 2L). Collectively, the *in vitro* cell experiments demonstrated that the resultant biohybrid bacteria EcN-IL-15/CR possess significant photothermal cytotoxicity and ICD-inducing capability.

### Tumor colonization of EcN-IL-15/CR

**Figure 3:**
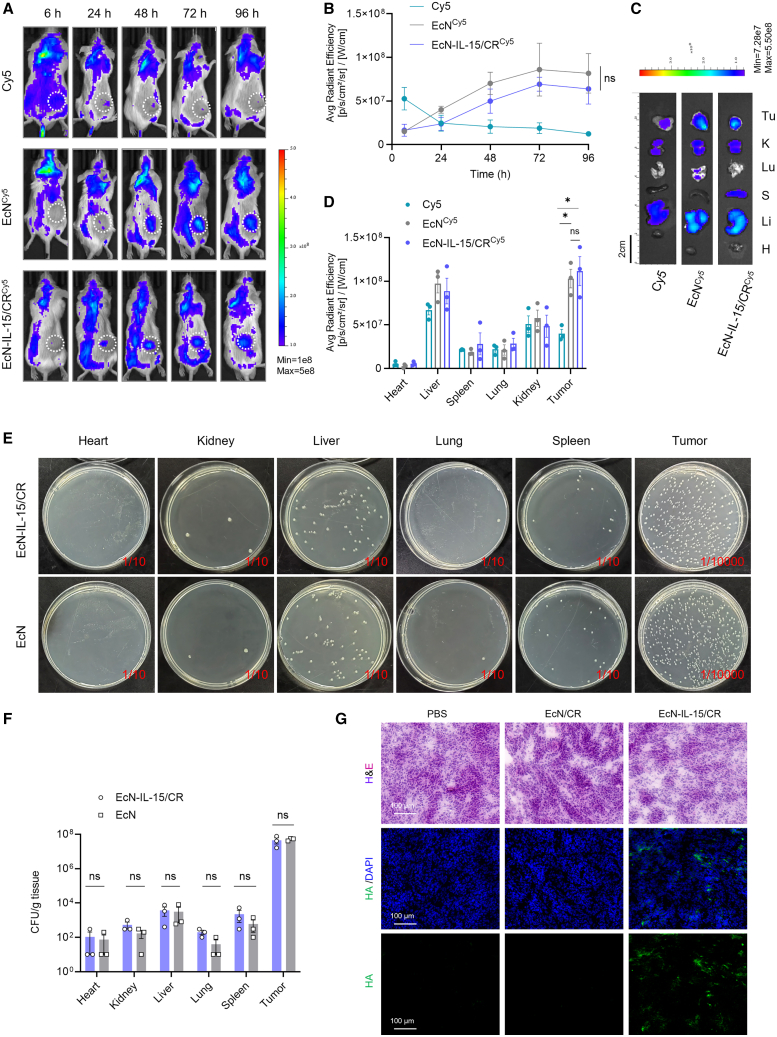
Bacterial colonization *in vivo* (A and B) *In vivo* optical living imaging of Colon26 tumor-bearing mice after intravenous injection of Cy5-labeled EcN or EcN-IL-15/CR bacteria (8 × 10^7^ CFU), or free Cy5. The scale (right) shows the upper and lower imaging thresholds. Quantification of Cy5 fluorescence signals for tumor accumulation at different time intervals; *n* = 3 biological replicates. (C and D) Fluorescence signals from major organs and tumors of tumor-bearing mice at 96 h post injection; *n* = 3 biological replicates. The scale (up) shows the upper and lower imaging thresholds. Scale bars, 2 cm. Tu, tumor; K, kidney; S, spleen; Lu, lung; Li, liver; H, heart. (E and F) Representative photographs (D) and quantification (E) of bacterial colonization in tumors and major organs. Colon26-bearing mice were intravenously injected with EcN or EcN-IL-15/CR (8 × 10^7^ CFU). After 48 h, tumors and organs were harvested for analysis; *n* = 3 biological replicates. (G) Immunofluorescence staining images of HA and H&E staining images, from adjacent sections. Colon26-bearing mice were intravenously injected with EcN/CR, EcN-IL-15/CR (8 × 10^7^ CFU), or PBS. After 48 h, tumors were harvested, embedded in OCT, and sectioned for analysis. Representative images of three tumors are shown. Scale bars, 100 μm. Data are presented as means ± SEM. *p* values were analyzed by two-way ANOVA with Tukey’s multiple comparisons test (B), one-way ANOVA with Tukey’s multiple comparisons test (D), and two-tailed unpaired Student’s t test (F). ∗*p* < 0.05; ns, not significant.

To assess the tumor-targeting capacity of EcN-IL-15/CR bacteria, EcN-IL-15/CR and EcN were labeled with cyanine 5 (Cy5)-NHS and intravenously injected into mice bearing Colon26 tumors and tracked over time using an IVIS spectrum imaging system. The fluorescence of both bacteria at the tumor sites increased progressively within 48 h, while free Cy5 decreased (Figures 3A and 3B). At 96 h post administration, the mice were euthanized to analyze fluorescence signals in various organs. The findings indicated that both EcN-IL-15/CR and EcN were primarily concentrated in the tumors and liver when compared with other organs (Figures 3C amd 3D). To quantify viable bacterial counts, tissue samples were homogenized and plated on Luria-Bertani agar, revealing predominant bacterial colonization in tumors over normal tissues (Figures 3E and 3F). A small number of bacterial colonies were also found in the kidney, spleen, and liver, but their numbers were over 1,000 times lower compared to those in the tumor tissues. We also found that, compared to the original EcN, the bacterial colonies of EcN-IL-15 and EcN-IL-15/CR in tumors did not show significant differences (Figure S7A). These observations suggest that the EcN-IL-15/CR engineering did not alter the tumor tropism of the bacteria. Besides, immunohistochemical (IHC) staining using an anti-*E. coli* antibody revealed a biodistribution pattern consistent with bacterial colony counts (Figure S7B).

Furthermore, we measured the concentrations of CR in various tissues 48 h post injection of EcN-IL-15/CR. Our results revealed that CR predominantly accumulated in the tumors and the liver, which likely plays a significant role in the metabolism of CR (Figure S8A). Although CR concentrations were high in the liver, bacterial colony counts were notably lower in the liver compared to the tumors (Figures 3E and 3F). This discrepancy may be attributed to phagocytosis or the clearance of bacteria by the liver, which typically filters and removes foreign particles from circulation.^39^^,^^40^ To evaluate the PTT potential of CR-modified bacteria, we irradiated the tumor site 48 h after injection with an 808 nm laser. The EcN-IL-15/CR group reached temperatures around 45°C, significantly higher than the separate administration of free CR-NHS and EcN-IL-15. However, there was no difference in photothermal performance between the two groups *in vitro*, as an equal amount of CR was irradiated in both cases (Figures S8B and S8C). These results highlight the advantage of CR-modified bacteria in enhancing *in vivo* delivery and achieving a more effective therapeutic response compared to free CR.

To assess the distribution of IL-15 delivered by bacteria in various tissues, we collected major tissue and serum samples from mice at 48 and 96 h post injection. The results showed that 48 h after injection of EcN-IL-15/CR, IL-15 levels in the kidneys and liver were significantly higher than those in the EcN/CR group but returned to baseline levels at 96 h. The transient elevation of IL-15 levels in the liver and kidneys may be attributed to drug metabolism or bacterial clearance over time. In contrast, IL-15 levels in tumor tissues remained elevated at both 48 and 96 h. No detectable increase in IL-15 levels was observed in the heart, lungs, spleen, or serum (Figure S8D). Moreover, IF staining of the HA tag validated the presence of HA-tagged IL-15 in the tumor sites (Figure 3G).

Taken together, these results provide solid evidence for the ability of engineered bacteria to colonize tumors and specifically deliver payloads intratumorally. This remarkable ability may be ascribed to the distinctive properties of a TME characterized by hypoxia and immunosuppression.^39^^,^^40^

### Photothermal EcN-IL-15/CR delays tumor progression in Colon26 colorectal cancer

**Figure 4:**
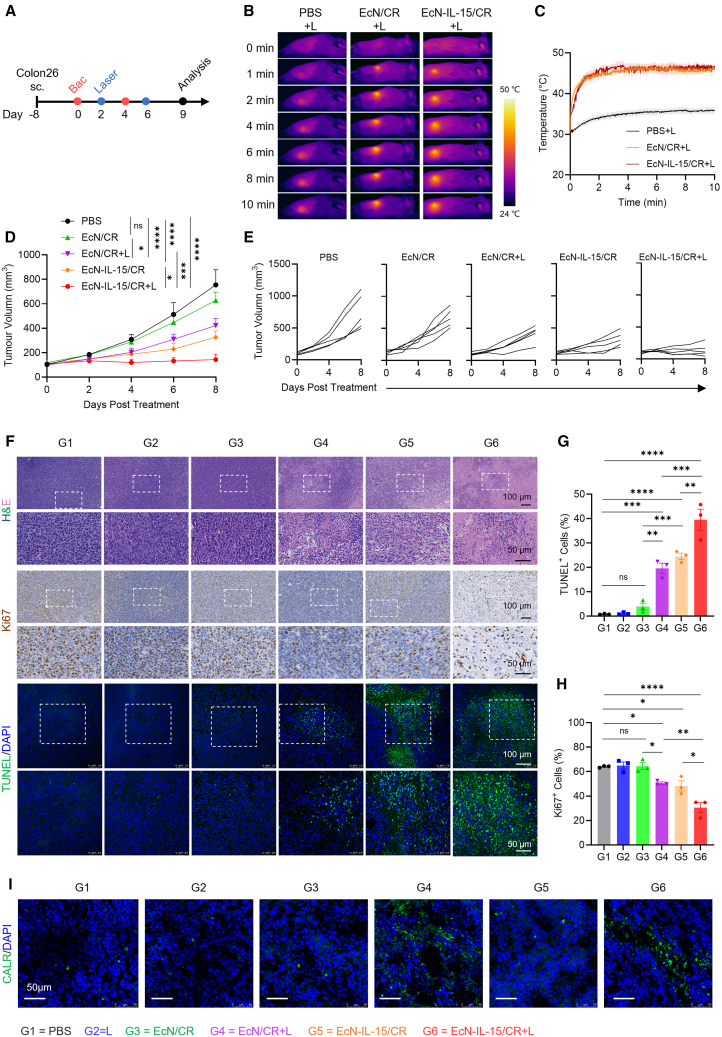
EcN-IL-15/CR suppresses tumor growth in Colon26 colorectal tumor (A) Treatment schedule of EcN-IL-15/CR in subcutaneous Colon26 tumor. (B and C) Representative thermal images and temperature variations of Colon26 tumor-bearing mice after intravenous injection of PBS, EcN/CR, or EcN-IL-15/CR, followed by 808 nm laser (1.2 W cm^−2^, 10 min); *n* = 3–4 biological replicates. (D and E) Colon26 tumor growth curves; *n* = 5 biological replicates. (F) Representative images of H&E-stained, Ki67-stained, and TUNEL-stained tumor sections from different groups, related to Figure S9. The images are representative of three mice, with ∼5 fields of view per sample. High-magnification images correspond to the areas marked by dotted white lines. Scale bars, 100 and 50 μm. (G and H) Quantification of TUNEL-positive cells and Ki67-positive cells in tumor sections from each group; *n* = 3 biological replicates. (I) Representative images of cell surface CALR staining in tumor slices. Scale bars, 50 μm. Tissues were stained with anti-CALR (green) and DAPI (blue), respectively. The images are representative of three mice, with ∼5 fields of view per sample. G1, PBS; G2, laser alone; G3, EcN/CR; G4, EcN/CR + laser; G5, EcN-IL-15/CR; G6, EcN-IL-15/CR + laser. Data are presented as means ± SEM. *p* values were analyzed by two-way ANOVA with Holm-Sidak’s multiple comparisons test (D), or one-way ANOVA with Holm-Sidak’s multiple comparisons test (G, H). ∗*p* < 0.05, ∗∗*p* < 0.01, ∗∗∗*p* < 0.001, ∗∗∗∗*p* < 0.0001; ns, not significant.

To investigate the *in vivo* antitumor efficacy of probiotically delivered IL-15 in combination with PTT, we established a subcutaneous Colon26 colorectal cancer model in BALB/c mice and followed the treatment schedule outlined in Figure 4A. The tumor temperature in PBS-injected mice experienced only minor changes (within 5°C) following 10 min of irradiation (Figures 4B and 4C). In contrast, mice in the EcN-IL-15/CR + L and EcN/CR + L groups exhibited a rapid and similar temperature rise at the tumor sites, reaching approximately 45°C within 3 min. A mild photothermal effect within the range of 42°C–45°C has been demonstrated to impose minimal harm on normal tissues while creating a conducive TME for eliciting robust immunological responses.^41^^,^^42^ While EcN/CR alone had no substantial impact on tumor growth, it delayed tumor growth following irradiation (Figures 4D and 4E). EcN-IL-15/CR alone also demonstrated therapeutic efficacy in the Colon26 model relative to the EcN/CR-treated group. Expectedly, EcN-IL-15/CR + L therapy displayed maximal antitumor activity among all groups (Figures 4D and 4E). In a more extensive animal study that included an additional irradiation-alone group (G2), similar findings were observed, further supporting the superior antitumor efficacy of EcN-IL-15/CR + L therapy (Figure S9). Given that light irradiation alone failed to demonstrate significant tumor suppression, it was excluded from subsequent *in vivo* investigations.

To gain a better understanding of treatment effectiveness, we performed H&E staining for tumor tissues, which revealed that EcN-IL-15/CR + L treatment caused large areas of cell death, characterized by the presence of nuclear crumpling and loss of cellular morphology (Figure 4F). Additionally, the terminal deoxynucleotidyl transferase-mediated dUTP nick end labeling (TUNEL) assay indicated the highest levels of cellular apoptosis in this group (Figures 4F and 4G). Moreover, IHC staining indicated a marked reduction of Ki67 expression in EcN-IL-15/CR + L-treated tumors (Figures 4F and 4H), suggesting a significant suppression in cell proliferation. Importantly, it is worth noting that tumors in both the EcN/CR + L and EcN-IL-15/CR + L groups exhibited increased CALR exposure compared to non-irradiated tumors (Figure 4I), providing evidence of ICD induction as a consequence of PTT.

To assess the viability and location of engineered bacteria in tumor tissues after treatment, tumor samples were collected 120 h after the final injection. The CFU quantification results showed that a large number of bacteria remained viable in the tumor core, with fewer at the margin (Figure S10A). The transmission electron microscopy results also showed that the distribution of *E. coli* within the tumors is predominantly concentrated on the tumor core, with their presence decreasing progressively toward the tumor margin (Figure S10B). This observation aligns with previous studies suggesting that *E. coli* selectively colonizes immune-privileged tumor cores and preferentially grows within the hypoxic and necrotic regions of tumors.^39^^,^^43^

To evaluate the biosafety of this therapeutic strategy, a comprehensive evaluation of safety indicators was conducted. The administration of bacteria led to a slight decrease in body weight, which returned to normal after the cessation of treatment (Figure S11A). Blood biochemistry analyses revealed that most indices in the treated groups stayed within normal ranges (Figures S11B and S11C). Furthermore, a thorough histopathological examination of the major organs did not reveal evident pathological alterations (Figure S11D). To further assess the potential toxicity induced by bacterial treatment, we conducted a time-course safety evaluation throughout a single treatment period (4 days). Daily monitoring of body temperature revealed that neither bacterial injection nor phototherapy caused any changes in body temperature (Figure S12A). Additionally, no significant alterations were detected in liver, kidney, or pancreas injury markers in tumor-bearing mice at 24, 48, and 96 h post injection (Figure S12B). Histopathological analysis of major organ tissues showed no visible structural damage or inflammatory cell infiltration (Figure S12C). Collectively, these results provide evidence that the bacterial therapy is generally well tolerated and safe.

### Photothermal EcN-IL-15/CR reprograms the TME

**Figure 5:**
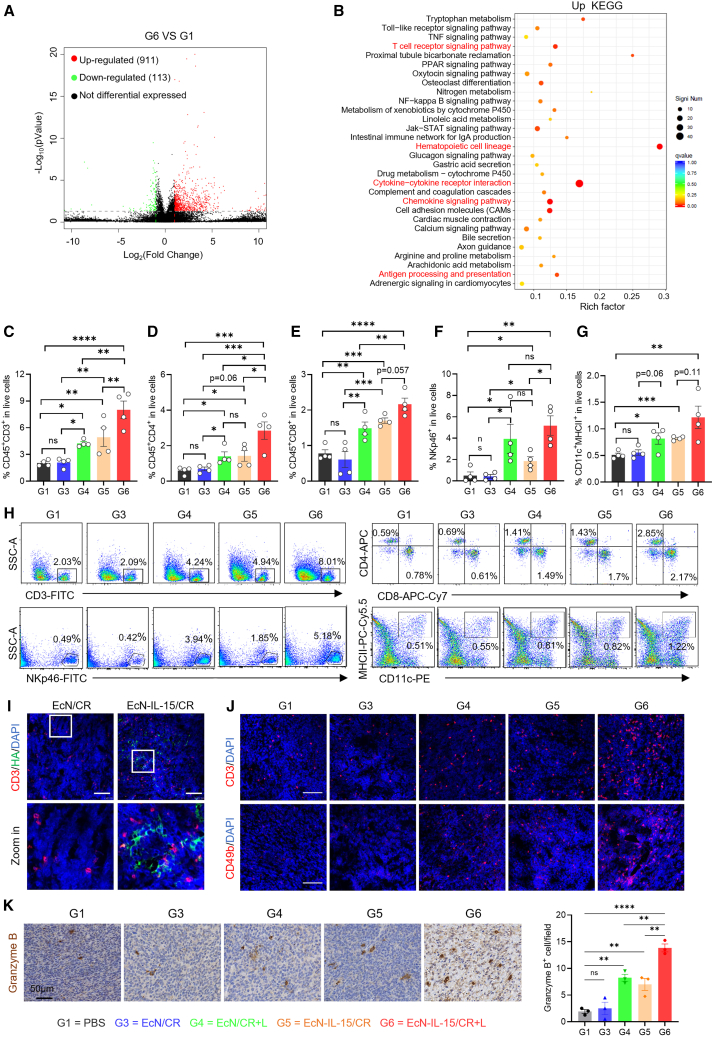
Photothermal EcN-IL-15/CR reshapes the TME to boost antitumor immunity (A and B) RNA-seq analysis of Colon26 tumors treated with EcN-IL-15/CR + L or PBS. (A) Volcano plot of differentially expressed genes. (B) KEGG enrichment analysis of upregulated genes; *n* = 3 biological replicates. (C–H) Flow cytometric analysis of immune cell infiltration in Colon26 tumors on day 9 post treatment. Quantification of CD3^+^ T cells (C), CD4^+^ T cells (D), CD8^+^ T cells (E), NK cells (F), and DCs (G). Colon26-bearing mice were treated as described in Figure 4A; *n* = 4 biological replicates. Experiment was repeated twice with similar results. (I) Representative IF images of HA tag and T cells in Colon26 tumor sections. HA tag, T cells, and nuclei were stained with anti-HA (green), anti-CD3 (red), and DAPI (blue), respectively. The images are representative of three mice, with ∼5 fields of view per sample. Scale bars, 100 μm. (J) Representative IF images of T cells and NK cells in Colon26 tumor sections. T cells, NK cells, and nuclei were stained with anti-CD3 (red), anti-CD49b (red), and DAPI (blue), respectively. The images are representative of three mice, with ∼5 fields of view per sample. Scale bars, 100 μm. (K) Representative IHC images and quantification of granzyme B in Colon26 tumor sections. The images are representative of three mice, with ∼5 fields of view per sample. Scale bars, 50 μm. G1, PBS; G3, EcN/CR; G4, EcN/CR + laser; G5, EcN-IL-15/CR; G6, EcN-IL-15/CR + laser. Data are presented as means ± SEM. *p* values were analyzed by two-tailed unpaired Student’s t test (D, F, G) or one-way ANOVA with two-stage linear step-up procedure of Benjamini, Krieger, and Yekutieli test (C, E), Holm-Sidak’s multiple comparisons test (K). ∗*p* < 0.05, ∗∗*p* < 0.01, ∗∗∗*p* < 0.001, ∗∗∗∗*p* < 0.0001; ns, not significant.

To explore the mechanisms of photothermal EcN-IL-15/CR on the Colon26 TME, we performed bulk RNA sequencing, which revealed 1,024 differentially expressed genes (911 upregulated, 113 downregulated) following EcN-IL-15/CR + L treatment (Figure 5A). Kyoto Encyclopedia of Genes and Genomes (KEGG) pathway analysis revealed profound alterations induced by EcN-IL-15/CR + L treatment (Figure 5B). Compared with PBS treatment, this therapeutic approach notably upregulated pathways associated with the stimulation of immune cell proliferation and their functional activation. These pathways included hematopoietic cell lineage, cytokine-cytokine receptor interaction, JAK-STAT signaling pathway, and T cell receptor signaling pathway. In addition, the chemokine signaling pathway and other immune-related processes, such as antigen processing and presentation, also displayed significant upregulation following EcN-IL-15/CR + L treatment. These alterations are likely related to the functions of IL-15 and the photothermal effects in the EcN-IL-15/CR + L group. However, we also observed an increase in the tryptophan metabolic pathway, suggesting potential associations with immune suppression or immune evasion.^44^

To investigate TME immune composition changes induced by photothermal EcN-IL-15/CR, we analyzed immunophenotypes in Colon26 tumors (Figure S13). In comparison with the PBS group, all experimental cohorts exhibited an increase in CD45^+^ tumor-infiltrating leukocytes to different extents, with the highest frequency recorded in the EcN-IL-15/CR + L group (Figures S14A and S14B). Although EcN/CR slightly promoted the infiltration of CD45^+^ leukocytes (*p* = 0.07), it did not significantly increase the frequency of CD3^+^ T or NK cells (Figures 5C–5F). In contrast, the administration of EcN-IL-15/CR alone resulted in a significant increase in the numbers of NK cells and CD3^+^ T cells, including CD4^+^ T cells and CD8^+^ T cells, relative to the PBS control (G5 vs*.* G1) and EcN/CR without engineered IL-15 (G5 vs*.* G3), respectively (Figures 5C–5F and 5H), indicating that delivery of IL-15 with engineered bacteria is an effective strategy for reshaping the TME. Immunostaining also demonstrated an accumulation of CD3^+^ T cells around the HA tag (Figure 5I). IL-15 stimulation promotes the survival and proliferation of NK and T cells by upregulating Bcl-2 and Ki67.^45^ Hence, these results suggest that bacterially delivered IL-15 contributes to enhancing the presence or expansion of both T and NK cell populations within the TME.

Photothermal treatment (EcN/CR + L) also resulted in an increase in intratumoral CD3^+^ T cells and NK cells compared to EcN/CR treatment alone or PBS (Figures 5C–5F and 5H). In addition, compared to PBS treatment, EcN/CR + L therapy upregulated the accumulation of DCs and macrophages, whereas EcN/CR alone did not (Figures 5G, S14C, S14D, and S15), which may be attributed to the PTT-triggered ICD effect. ICD occurs with CALR exposure on dying cell surfaces, providing an “eat me” signal to phagocytic cells, while the release of ATP acts as a “find me” signal, both of which together promote DC and macrophage infiltration at the tumor sites.^46^ Intriguingly, an obvious increase in DCs was also observed after EcN-IL-15/CR treatment (G5 vs. G3; Figure 5G), probably because of the conversion of monocytes to DCs by IL-15 stimulation.^47^^,^^48^ However, despite the significant changes in other immune cell populations, the ratio of M1/M2 macrophages exhibited no significant variation across all groups (Figures S14E–S14H), and further research is needed to elucidate the underlying mechanisms.

As expected, the EcN-IL-15/CR + L group exhibited the greatest increase in infiltrating NK and T cells (Figures 5C–5F, 5J, and S16), suggesting a synergistic effect between IL-15 and PTT. Additionally, granzyme B^+^ cells were more abundant in tumors treated with EcN-IL-15/CR + L than in EcN-IL-15/CR or EcN/CR + L groups (Figure 5K), indicating enhanced lymphocyte cytotoxicity for tumor elimination.^45^^,^^49^

Collectively, these findings provide valuable insights into TME reprogramming and complex interplay between immune cells in response to the photothermal EcN-IL-15/CR therapeutic approach.

Given that IL-15 regulates the maintenance and proliferation of CD8^+^ and CD4^+^ memory T cells,^37^^,^^50^^,^^51^ we investigated whether EcN-IL-15/CR + L treatment could induce the generation of memory T cells. This was accomplished by the adoptive transfer of splenocytes from EcN-IL-15/CR + L-treated tumor-bearing mice to naive recipients (Figure S17A). Expectedly, splenocytes from the EcN-IL-15 + L group significantly delayed tumor growth and prolonged survival compared to those from the PBS group (Figures S17B–S17D).

To further elucidate the mechanism underlying the protection conferred by EcN-IL-15/CR + L treatment, we analyzed the immunophenotype of splenocytes obtained from mice treated with EcN-IL-15/CR + L or PBS. The T effector memory (Tem) cells, located in both lymphoid and non-lymphoid tissues, primarily contribute to stronger cytotoxic lytic functions to facilitate pathogen clearance upon re-exposure to tumor antigens relative to the T central memory (Tcm) cells.^51^ As depicted in Figures S17E–S17G, the administration of EcN-IL-15/CR + L resulted in an expansion of Tem subsets and a concurrent reduction in Tcm subsets in both CD8^+^ and CD4^+^ T cells. This evidence suggests that EcN-IL-15/CR + L treatment promotes Tem establishment and these memory populations provide passive immunity to delay tumorigenesis in naive mice.

### EcN-IL-15/CR + L synergizes with ICB and promotes long-term immunological memory

**Figure 6:**
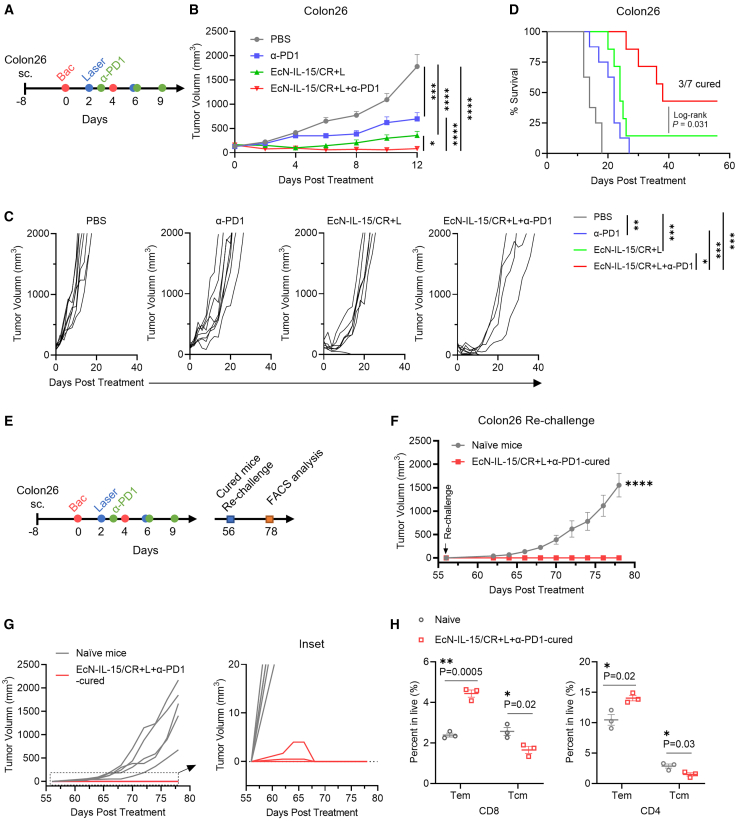
Photothermal EcN-IL-15/CR synergizes with PD-1 blockade to promote Colon26 tumor rejection and long-term immunological memory (A) Treatment schedule of EcN-IL-15/CR + L combined with anti-PD-1 therapy in subcutaneous Colon26 tumor. (B–D) Tumor growth and survival analysis; *n* = 7–8 biological replicates. (E) Schematic of the tumor re-challenge experiment to assess the long-term immunological memory triggered by EcN-IL-15/CR + L and PD-1 blockade. (F and G) Tumor growth curves of the rechallenged mice; *n* = 3 for cured mice, *n* = 5 for naive mice. (H) Quantification of Tem and Tcm subsets in spleens of rechallenged mice; *n* = 3 biological replicates. Data are presented as means ± SEM. *p* values were analyzed by two-way ANOVA with Holm-Sidak test (B), Sidak’s (F) multiple comparisons test, Mantel-Cox log rank test (D), or two-tailed unpaired Student’s t test (H). ∗*p* < 0.05, ∗∗*p* < 0.01, ∗∗∗*p* < 0.001, ∗∗∗∗*p* < 0.0001.

EcN-IL-15/CR + L remodels the TME by recruiting and expanding T/NK cells, which often express exhaustion markers like PD-1.^52^ Combining it with ICBs could reverse T cell exhaustion and enhance antitumor efficacy. We tested this hypothesis by treating Colon26 tumors with EcN/IL-15 + L and PD-1 blockade therapy (Figure 6A). EcN/IL-15 + L combined with anti-PD-1 antibodies significantly suppressed tumor growth, achieving complete tumor rejection in 43% of mice (3/7), versus 14% (1/7) with EcN/IL-15 + L alone. Anti-PD-1 alone had a modest effect without complete eradication (Figures 6B and 6C). This combination also markedly extended survival (Figure 6D), indicating that EcN/IL-15 + L enhances the efficacy of ICB immunotherapy.

Following a 40-day observation period after tumor eradication to ensure the absence of tumor recurrence, we rechallenged the mice whose tumors were completely eliminated after combinational therapy with the same cancer cells (1 × 10^6^ Colon26 cells/mouse) in opposite sites to the primary tumor location, in parallel with naive age-matched control mice (Figure 6E). All rechallenged mice rejected the tumors, with partial tumors initially growing but ultimately regressing (Figures 6F and 6G), indicating the establishment of long-term immunological memory. We also conducted flow cytometry to assess alterations in memory T cells in the spleens of the rechallenged mice. Intriguingly, a noticeable transition of Tcm to the Tem phenotype was observed in both CD8^+^ and CD4^+^ T cells among long-term surviving mice in response to EcN/IL-15 + L in conjunction with an anti-PD-1 antibody, compared to naive controls (Figures 6H and S18). Consequently, these findings offer sufficient evidence that robust and enduring immunological memory is induced by the combination of EcN/IL-15 + L and checkpoint blockade.

Tumors unresponsive to immune checkpoint inhibitors (ICIs) remain a major hurdle in immunotherapy. Although Colon26 tumors respond well to PD-1 antibodies, they may not accurately represent ICI-resistant tumors. To better assess our bacterial therapy, we tested its antitumor efficacy in an ICI-unresponsive model. We employed Lewis lung carcinoma (LLC) tumors, which are known to exhibit T cell exclusion.^53^^,^^54^ To assess the efficacy of the EcN-IL-15/CR + L treatment, photothermal treatment was performed within a mild-temperature range (Figures S19A and S19B). Treatment with EcN-IL-15/CR + L alone significantly extended survival compared to the PBS control. Notably, while anti-PD-1 antibodies alone did not improve survival, their addition to the EcN-IL-15/CR + L regimen resulted in further significant improvements in survival outcomes (Figures S19C–S19E). These results highlight that EcN-IL-15/CR + L is effective in treating “cold” tumors, those that are resistant to ICI therapy, and can induce a response to ICI treatment when combined with anti-PD-1 antibodies. This finding suggests that our therapeutic approach has the potential to overcome resistance in tumors traditionally unresponsive to immunotherapy.

### EcN-IL-15/CR + L combined with ICB reduces tumor metastasis

**Figure 7:**
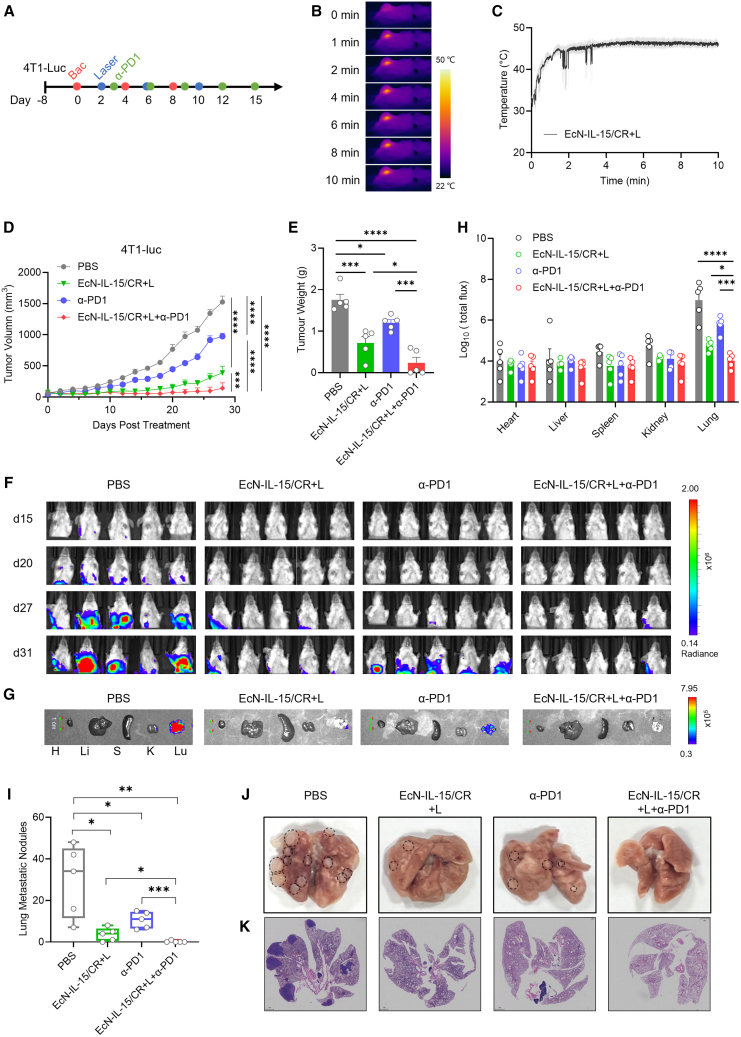
Photothermal EcN-IL-15/CR synergizes with PD-1 blockade to reduce tumor metastasis in an orthotopic breast cancer model (A) Treatment schedule of EcN-IL-15/CR + L combined with anti-PD-1 therapy in the 4T1-luc orthotopic breast cancer. (B and C) Representative thermal images (B) and temperature variations (C) of 4T1-luc tumor-bearing mice exposed to 808 nm laser irradiation (1.2 W cm^−2^, 10 min) after intravenous injection of EcN-IL-15/CR; *n* = 3 biological replicates. (D–F) Tumor progression and lung metastasis in the 4T1-luc model; *n* = 5 biological replicates. (G and H) Representative bioluminescence images and quantification of metastatic foci in major organs dissected on day 31. Scale bar, 1 cm (G). The scale (right) shows the upper and lower bioluminescence imaging thresholds. Bioluminescence photon flux from each organ was quantified; *n* = 5 biological replicates. H, heart; Li, liver; S, spleen; K, kidney; Lu, lung with metastatic nodules. (I–K) Assessment of lung metastasis on day 31; *n* = 5 biological replicates. Data are presented as means ± SEM. *p* values were analyzed by two-way ANOVA with Tukey’s multiple comparisons (D) or one-way ANOVA with Holm-Sidak’s multiple comparisons test (E, H) and two-tailed unpaired Student’s t test (I). ∗*p* < 0.05, ∗∗∗*p* < 0.001, ∗∗∗*∗p* < 0.0001.

Clinically, approximately 90% of cancer-associated deaths result from metastasis.^55^ To investigate the anti-metastatic potential of combining EcN-IL-15/CR + L with ICBs, we established an orthotopic breast cancer model by injecting 4T1-luc cells into the mammary fat pad of female BALB/c mice (Figure 7A). Tumors in mice that received EcN-IL-15/CR were irradiated 48 h post injection, also resulting in mild hyperthermia (∼45°C; Figures 7B and 7C). Similar to the Colon26 model, the treatment efficacy of EcN-IL-15/CR + L with anti-PD-1 was superior to that of EcN-IL-15/CR + L and anti-PD-1 administered individually, resulting in complete tumor rejection in 40% of treated mice (2/5; Figures 7D, 7E, and S20A).

As tumors progressed, all mice in the PBS and anti-PD-1 groups developed marked lung metastases by day 31, while 80% of EcN-IL-15/CR + L-treated mice (4/5) exhibited only mild metastatic signals (Figure 7F). Impressively, the combinatorial regimen of EcN-IL-15/CR + L and anti-PD-1 showed a significant reduction in metastatic signals, with only 20% of the mice (1/5) showing limited metastatic progression (Figures 7F–7H). Quantification of lung tumor nodules and histological analysis further confirmed the therapeutic benefit (Figures 7I–7K and S20B). Together, these results demonstrate the prominent antitumor potential of this combined treatment strategy in this aggressive and low-immunogenicity tumor model.

## Discussion

Here, we developed a protease-sensitive IL-15-engineered bacterial system for tumor-targeted therapy. (1) Systemically administered *E. coli* delivered IL-15 into tumors due to its natural tumor tropism. (2) Tumor-enriched proteases cleaved membrane-bound IL-15, helping to minimize off-tumor immune activation. (3) CR modification enabled mild photothermal effects that triggered ICD and enhanced IL-15 efficacy. (4) This bacterial hybrid reprogrammed the TME, improved immunotherapy response, and induced durable antitumor immunity against both primary and metastatic tumors.

Engineered bacteria have shown promise in cancer therapy for drug delivery and TME modulation.^43^^,^^56^^,^^57^^,^^58^^,^^59^ Bacterial delivery of cytokines has demonstrated TME reprogramming and antitumor effects,^60^^,^^61^ as seen in studies using attenuated *Salmonella* to express flagellin B and IL-15 fusion proteins.^60^ However, their reliance on daily L-arabinose injections is limited by uneven inducer penetration into tumors. Our strategy addresses this by pre-inducing IL-15 expression in EcN, with surface-tethered, protease-cleavable IL-15 ensuring tumor-specific release. Compared to other bacteria, EcN offers a better safety profile and robust genetic tools.^56^^,^^59^^,^^62^^,^^63^^,^^64^^,^^65^^,^^66^ In a clinical study (NCT04167137), the engineered EcN strain SYNB1891, expressing a stimulator of interferon genes agonist, was tested in refractory advanced cancers via intratumoral injections, alone or with atezolizumab.^67^ The regimen was safe and well tolerated, with some cases of cytokine release syndrome. While stable disease was observed in some PD-1/L1-refractory patients, overall efficacy was limited. Moreover, intratumoral injection restricts its clinical applicability, highlighting the need for alternative EcN-based strategies. This strategy described in our study allows for the delivery of immunomodulatory agents and photothermal agents to the TME, harnessing the benefits of targeted therapy while improving therapeutic outcomes.

The concept of protease-sensitive cytokine bacteria described in this study opens new avenues for bacterial engineering. Various techniques for inducible gene expression and payload release in bacteria include chemical inducers, biological methods, and physical modalities.^22^ Chemical inducers like arabinose and IPTG lack site specificity. Biological methods, such as promoter engineering and sense-and-respond genetic circuitry, autonomously control bacterial behavior to manage the growth and payload release.^23^^,^^59^^,^^65^^,^^68^ However, designing these systems can be complicated. Physical modalities like focused ultrasound and light radiation offer localized control but are limited by irradiation position and timing.^39^^,^^69^ Inspired by probody/pro-drug strategies,^70^^,^^71^^,^^72^^,^^73^ we engineered bacteria to express membrane-tethered cytokines via a protease-cleavable linker. Tumor-associated proteases trigger cytokine release specifically in the TME. This approach enables controllable *in vitro* expression and tumor-targeted *in vivo* release without external stimuli.

Beyond bacterial engineering, our study shows that combining probiotic IL-15 with PTT enhances anti-PD-1 efficacy in both “hot” (Colon26^74^) and “cold” (4T1, LLC^41^^,^^75^) tumors. Notably, PTT increased antigen-presenting cell (APC) infiltration, including macrophages and DCs, into the TME. Crucially, the combination of probiotic IL-15 and PTT synergistically elevated the presence of various immune cells including T cells, NK cells, DCs, and macrophages. This shift likely results from the differentiation, development, and recruitment of peripheral immune cells,^76^ as suggested by the upregulation of the hematopoietic cell lineage and chemokine signaling pathways in the KEGG analysis. Moreover, the upregulation of the antigen processing and presentation pathway suggests that the therapy enhances the ability of APCs, likely DCs and macrophages, to process and present tumor-specific antigens. This, in turn, activates T cells and triggers an adaptive immune response against the tumor.^77^ Furthermore, the upregulation of the cytokine-cytokine receptor interaction, JAK-STAT signaling, T cell receptor signaling, Toll-like receptor signaling, TNF signaling, and nuclear factor κB signaling pathways further supports the hypothesis that EcN-IL-15/CR + L treatment promotes the formation of an immunoreactive TME, which directly contributes to enhanced antitumor immunity.^78^

Although many PTT studies have been limited to preclinical models, the clinical use of photothermal ablation has been demonstrated. For example, a phase 1 trial employed gold nanoshells with interstitial laser treatment for focal photothermal ablation of prostate tumors,^79^ and Akalux, an NIR photoimmunotherapy agent, has been approved in Japan in 2020 for advanced or recurrent head and neck cancer.^80^ Studies indicate that NIR laser light can penetrate soft tissues up to 1–2 cm.^81^ While this may not suffice for targeting deeply located tumors, the use of optical fibers inserted directly into the tumor or irradiation devices embedded in endoscopic and laparoscopic systems enables internal photothermal treatment for various cancers.^82^ This demonstrates that PTT is not limited to surface lesions and can be adapted for deeper, more challenging tumor sites. This further underscores the clinical potential of photothermal therapies using bacteria.

While the potential for immune responses against IL-15 and EcN is a valid concern, it is crucial to highlight that both components are unlikely to induce robust neutralizing antibody responses under our experimental conditions. In our study, IL-15 is murine derived, which significantly minimizes the risk of eliciting neutralizing antibodies in mouse models. Moreover, EcN, originally isolated from humans and widely recognized as a probiotic, has been extensively utilized in clinical settings, particularly for the treatment of gastrointestinal disorders. Studies have shown that EcN can modulate the immune system in a way that promotes tolerance rather than aggressive immune activation, which may reduce the risk of neutralizing antibody production.^83^^,^^84^ However, the risk of immune-mediated neutralization cannot be entirely ruled out. Therefore, further investigation into potential antidrug immune responses is warranted to comprehensively evaluate the therapeutic potential of this approach.

### Limitations of the study

Although EcN-IL-15/CR + L showed tolerable systemic toxicity in mice, further safety evaluation is needed due to greater human sensitivity to bacteria and endotoxins. As discussed, assessing antidrug immune responses is essential before clinical translation. Additionally, further studies are required to determine whether similar effects can be achieved in human tumors treated with EcN-IL-15/CR + L.

## Resource availability

### Lead contact

Further information and requests for resources and materials should be directed to the lead contact, Jigang Wang (wangjigang@u.nus.edu).

### Materials availability

This study did not generate new unique reagents.

### Data and code availability

• The RNA-seq data have been deposited and made publicly available in the Genome Sequence Archive (GSA) at the National Genomics Data Center (accession number GSA: CRA024724).
• This paper does not report original code.
• Any additional information required to reanalyze the data reported in this paper is available from the lead contact upon request.

## Acknowledgments

This work was supported by grants from the 10.13039/501100001809National Natural Science Foundation of China (U24A20798; 82373775; 82404492), the 10.13039/501100012166National Key Research and Development Program of China (2022YFC2303600), the Key Special Project of Strategic Science and Technology Innovation Cooperation from National Key R&D Program (2023YFE0204500), 10.13039/501100021171Basic and Applied Basic Research Foundation of Guangdong Province (2023A1515110034), 10.13039/501100002858China Postdoctoral Science Foundation (2022M712189), Shenzhen Medical Research Fund (D2403013 and B2302051), 10.13039/501100010877Shenzhen Science and Technology Innovation Committee (RCBS20210706092213007), the Scientific and Technological Innovation Project of 10.13039/501100005892China Academy of Chinese Medical Sciences (CI2023D003, CI2021B014, and CI2023D008), the CACMS Innovation Fund (CI2023E002, CI2021A05101, and CI2021A05104), the Science and Technology Foundation of Shenzhen (Shenzhen Clinical Medical Research Center for Geriatric Diseases), the 10.13039/501100010877Shenzhen Science and Technology Innovation Committee (SZSTI) (RCYX20221008092950121), the Natural Science Foundation of Top Talent of SZTU (GDRC202125), the 10.13039/501100010877Shenzhen Science and Technology Innovation Commission (JCYJ20200109120205924), Shenzhen Key Medical Discipline Construction Fund (SZXK046), International Science and Technology Cooperation for Shenzhen Technology Innovation Plan (GJHZ20240218114508015), Shenzhen Governmental Sustainable Development Fund (KCXFZ20201221173612034), Shenzhen key Laboratory of Kidney Diseases (ZDSYS201504301616234), Shenzhen Fund for Guangdong Provincial High-level Clinical Key Specialties (no. SZGSP001), Shenzhen People’s Hospital Fund (SYWGSCGZH202405; SYWGSJCYJ202201), and Natural Science Foundation of Top Talent of SZTU (GDRC202125).

## Author contributions

H.W. designed and performed most experiments, collected and analyzed the data, and drafted the manuscript. L.Z. designed and performed most experiments, analyzed experiments involving flow cytometry analysis, and revised the manuscript. C.Y. and L.J. provided technical advice and analyzed the data. Y.D. provided CR-NHS dye. R.Z., H.L., X.X., G.S., J.Y., Y. Li, H.Y., J.C., G.Z., L.Y., T.G., and H.J. assisted with *in vitro* experiments and animal experiments. Y. Liu, X.W., Z.L., and J.W. conceived and supervised the study.

## Declaration of interests

The authors declare no competing interests.

## STAR★Methods

### Key resources table

**Table:** 

REAGENT or RESOURCE	SOURCE	IDENTIFIER
**Antibodies**		

Anti-HA	Cell Signaling Technology	Cat#3724; RRID: AB_1549585
anti-PD1 antibody (clone RMP1-14)	Bioxcell	Cat# BE0146; RRID: AB_10949053
anti-mouse CD16/CD32 antibody	Bioxcell	Cat# BE0307; RRID: AB_2736987
anti-CD45-PECF594	BD Biosciences	Cat#562420; RRID: AB_11154401
anti-CD3-FITC	BD Biosciences	Cat#553062; RRID: AB_394595
anti-CD4-APC	Biolegend	Cat#100412; RRID: AB_312696
anti-CD8a-APC-Cy7	BD Biosciences	Cat#557654; RRID: AB_396769
anti-CD11b-APC-Cy7	Biolegend	Cat#101226; RRID: AB_830641
anti-F4/80-APC	Biolegend	Cat#123116; RRID: AB_893481
anti-NKp46-FITC	Biolegend	Cat#137606; RRID: AB_2298210
anti-CD45-BV570	Biolegend	Cat#103136; RRID: AB_10898325
anti-CD8a-PE	Biolegend	Cat#100708; RRID: AB_312747
anti-CD206 (MMR)-PE-Cy7	Biolegend	Cat# 141720; RRID: AB_2562247
anti-MHCII-PerCp-Cy5.5	Biolegend	Cat# 107626; RRID: AB_2191071
anti-CD62L-BV421	Biolegend	Cat#104435; RRID: AB_10900082
anti-CD44-AF700	Biolegend	Cat#103026; RRID: AB_493712
anti-CD3	Abcam	Cat#ab5690; RRID: AB_305055
anti-CD3	Biolegend	Cat#100202; RRID: AB_312658
anti-CD8	BD Biosciences	Cat#550281; RRID: AB_2275792
anti-CD49b	BD Biosciences	Cat#553855; RRID: AB_395091
anti-E.coli	Abcam	Cat#ab137967; RRID: AB_2917966
anti-CD11c	BD Biosciences	Cat#550283; RRID: AB_393578
anti-CALR	Abcam	Cat# ab92516; RRID: AB_10562796
anti-HMGB1 (Abcam, ab18256).	Abcam	Cat# ab18256; RRID: AB_444360

**Bacterial and virus strains**		

Escherichia coli Nissle 1917	Gift from Southern Medical University	N/A

**Chemicals, peptides, and recombinant proteins**		

MMP2	Sino Biological	Cat#10082-HNAH
MMP9	Sino Biological	Cat#10327-HNAH
uPA	Sino Biological	Cat#10815-H08H
*p*-aminophenylmercuric acetate	Sigma	Cat#A9563
IL-15	Abclonal	Cat#RP01676
EDC	Sigma	Cat# 39391
N-hydroxysuccinimide (NHS)	Aladdin	Cat# H109330
CR-NHS	This paper	N/A
Cy5-NHS	Ruixi Biotechnology Co.	Cat#R-SX-997

**Critical commercial assays**		

calcein-AM and PI (live/dead)	Beyotime	Cat#C2015
IFN-γ ELISA kit	Proteintech	Cat#KE10094
IL-6 ELISA kit	Proteintech	Cat#KE10007
Tissue enzymatic digestion kit	RWD Life Science Co., LTD	Cat#DHTE-5001
CCK8 Kit	Epizyme	Cat#CX001M
CFSE Cell Proliferation Kit	Invitrogen	Cat#C34570

**Deposited data**		

RNA-seq	This paper	GSA: CRA024724

**Experimental models: Cell lines**		

Colon26	Gift from Southern Medical University	N/A
4T1-Luc	FuHeng	Cat#FH1114
MC38	FuHeng	Cat#FH0644
LLC	FuHeng	Cat# FH0325

**Experimental models: Organisms/strains**		

Mouse: BALB/c.	GemPharmatech	N/A
Mouse: C57BL/6JNarl	GemPharmatech	N/A

**Recombinant DNA**		

pNeae2 plasmid	Addgene	N/A

**Software and algorithms**		

GraphPad Prism version 8	GraphPad	https://www.graphpad.com/
Biorender	Biorender	https://www.biorender.com/
FlowJo 10.6.5	FlowJo, LLC	https://www.flowjo.com/
Living Image software	PerkinElmer	N/A

### Experimental model and study participant details

#### Cell lines

MC38, Colon26, and 4T1 cells were cultured in RPMI 1640 medium (Gibco) supplemented with 10% fetal bovine serum (Gibco) and 1% penicillin-streptomycin (Gibco) at 37°C with 5% CO_2_. LLC cells were cultured in DMEM medium (Gibco) under the same conditions.

#### Animals

C57BL/6 and BALB/c Mice (6–12 weeks old) were obtained from GemPharmatech Company and maintained under specific pathogen-free (SPF) conditions with 12-h light/dark cycles. All experiments and euthanasia procedures were conducted with the approval of the Institutional Animal Care and Use Committee (IACUC) of Shenzhen People’s Hospital (AUP-220501-LZJ-0595-01).

### Method details

#### Plasmid construction

The pNeae2 plasmid was obtained from Addgene. The remaining constructs were designed by the authors and synthesized by GENEWIZ. To construct IL-15 surface-expressing bacteria, the DNA sequence of IL-15 and protease cleavable sequence were inserted into the pNeae2 vector, and the resulting plasmid was named pNeae2-IL-15 (Table S1). Plasmids pNeae2-IL-15 and empty vector (pNeae2) were transformed into competent EcN cells and stored in chloramphenicol-containing media (50 μg mL^−1^). The resulting engineered bacteria were named EcN-IL-15 and EcN, respectively. EcN-IL-15 bacteria were cultured until optical density reached 0.3 to 0.5 (OD600). Then, isopropyl β-D-1-thiogalactopyranoside (IPTG, 0.5 mM) was added and cultured at 37°C for 6 h. Flow cytometry and western blot were used to detect the HA tag expression on the outer membrane of EcN-IL-15.

#### Cleavage of bacteria by recombinant proteases

Recombinant human MMP2, MMP9, and uPA were purchased from Sino Biological. MMP2 and MMP9 were activated using 1 mM *p*-aminophenylmercuric acetate (APMA, Sigma) according to the product datasheet of each MMP. Following activation, MMPs were diluted to various concentrations in an assay buffer containing 150 mM NaCl, 10 mM CaCl_2_, 50 mM Tris-HCl, and 0.05% Brij-35 (pH 7.5). uPA, provided as an active enzyme, was diluted in an assay buffer containing 150 mM NaCl, 50 mM Tris, and 0.01% (v/v) Tween 20 (pH 8.5). EcN-IL-15 bacteria (4 × 10^8^ CFU) was suspended in 1 mL protease solution and incubated at 37°C. Protein cleavage was confirmed by western blot detection of HA tag in bacteria supernatant. The cell proliferation assay was used to detect the bioactivity of EcN-IL-15. Mouse splenocytes were harvested, labeled with carboxyfluorescein diacetate succinimidyl ester (CFSE), and stimulated for 72 h with supernatant after MMP2 cleavage of EcN or EcN-IL-15, followed by FACS analysis and cell counting kit-8 (CCK8) assay.

#### Cleavage of bacteria by tumor and normal tissues

Tissues were homogenized in PBS, and the supernatant was obtained by centrifugation at 10,000 × g for 20 min. Protein concentration was determined using a BCA kit, and supernatants were stored at −80°C. For cleavage assays, tissue lysates (2 mg/mL) were incubated with EcN-IL-15 bacteria (4 × 10^8^ CFU) and MMP assay buffer at 37°C for the indicated durations. Protein cleavage was confirmed by western blot detection of HA tag in bacteria supernatant.

#### Preparation of photothermal bacteria

CR-NHS was synthesized as previously described.^38^ Briefly, croconium dye (150 mg, 0.28 mmol), EDC (130 mg, 0.85 mmol), and N-hydroxysuccinimide (NHS, 97 mg, 0.85 mmol) in DMF (10 mL) were mixed and stirred at room temperature overnight. The organic solvent was removed under reduced pressure. The residue was dissolved in ethyl acetate (20 mL), followed by washing with dH_2_O (3 × 30 mL), dried with Na_2_SO_4_, and concentrated to obtain a navy blue solid (CR-NHS).

EcN-IL-15 bacteria were pre-induced with 0.5 mM IPTG for 6 h, followed by washing three times with PBS before proceeding with the reaction. The preparation of photothermal bacteria was based on the reaction of the NHS ester and amines on the bacteria’s surface. Typically, CR-NHS (100 μg) in 2 mL PBS was added to bacteria pellets (4 × 10^8^ CFU) and incubated at 37°C for 30 min, followed by washing twice with PBS.

#### Photothermal performance

To explore the photothermal conversion effect of the fabricated photothermal bacteria, the EcN-IL-15 or EcN-IL-15/CR suspension (4 × 10^8^ CFU/mL) was exposed to 808 nm laser irradiation (1.2 W cm^−2^) for 10 min. To examine the influences of different power densities on heating, the EcN-IL-15/CR suspension (4 × 10^8^ CFU/mL) was exposed to 808 nm laser irradiation at 0.5, 0.8, and 1.2 W cm^−2^ for 10 min. The thermal images and temperature variations of the bacteria suspensions were recorded by a thermal imaging camera.

#### Cell viability assay

Colon26 cells or 4T1 cells (4 × 10^3^ cells) were seeded into a 96-well plate and cultured overnight. Different amount of EcN-IL-15/CR or EcN/CR solution was added to the cells and incubated for 1 h at 37°C. Then the cells were treated with or without 808 nm laser irradiation (1.5 W cm^−2^, 10 min) and incubated at 37°C for 1 h. The cells were then washed twice with RPMI 1640 medium containing antibiotics (2%) and further incubated for another 48 h. The cell viability was detected by CCK8 assay.

#### Live/dead cell staining assay

Colon26 cells (1 × 10^4^ cells) were seeded into a 96-well plate and cultured overnight. EcN-IL-15/CR or EcN/CR solution (2 × 10^8^ CFU/mL) was added to the cells and incubated for 1 h at 37°C. Then the cells were treated with or without 808 nm laser irradiation (1.5 W cm^−2^, 10 min) and incubated at 37°C for 1 h. The cells were then washed twice with RPMI 1640 medium containing antibiotics (2%) and further incubated for another 24 h. After incubation, the cells were washed with PBS, stained by calcein-AM and PI (live/dead), and immediately observed by a confocal laser scanning microscopy (Leica SP8, Germany).

#### Animal models

To generate the subcutaneous tumor models, MC38 (1 × 10^6^ cells/100 μL), LLC (2 × 10^6^ cells/100 μL) or Colon26 (1 × 10^6^ cells/100 μL) cells were implanted into the right dorsal flank of C57BL/6 mice or BALB/c mice. To generate the orthotopic murine breast cancer model, 4T1-luc cells (5 × 10^5^ cells/50 μL) were injected into the breast pad of each female BALB/c mouse, as described previously.^85^ Tumor-bearing mice with tumor volume reaching 100–150 mm^3^ (MC38), or 50–100 mm^3^ (Colon26, 4T1, LLC) were randomly assigned to different groups and subsequently subjected to various therapeutic interventions. Tumors were measured with a caliper every 2 days, and tumor volume was calculated by the following formula: 0.5 × length × width^2^. The metastases of 4T1-luc tumors were monitored by an IVIS imaging system (PerkinElmer, USA). In accordance with the IACUC protocol, individual mice were euthanized if the tumor exceeded the 2000 mm^3^ humane limit or had a 20% weight loss, or if the tumor was necrotic, ulcerated, bleeding, or impaired the nutrition or health of the mice.

For complete responder mice, the mice were monitored for at least 40 days post-tumor regression and were rechallenged by subcutaneous implantation of Colon26 cells (1 × 10^6^ cells/100 μL) on the left dorsal flank alongside naive age-matched controls. At the endpoint of the study, the spleens of mice were harvested for immune phenotyping analysis. This experiment was performed once.

#### Animal treatments

To evaluate the antitumor efficacy of EcN-IL-15, MC38-bearing mice were randomly divided and intravenously treated with PBS, EcN-IL-15 (4 × 10^7^ CFU), or EcN (4 × 10^7^ CFU) every 4 days for a total of two injections. To compare the toxicity and efficacy of EcN-IL-15 and recombinant IL-15, MC38-bearing mice were intravenously treated with PBS, EcN-IL-15 (4 × 10^7^ CFU, two injections in total), or recombinant mouse IL-15 protein (four injections in total). High-dose IL-15 (H) was administered at 10 μg/mouse, while low-dose IL-15 (L) was given at 5 μg/mouse. Blood samples were collected on day 9 for biochemical tests and ELISA analysis. To assess the antitumor efficacy of EcN-IL-15/CR in combination with PTT, Colon26-bearing mice were randomly assigned to the following groups: PBS, L (808 nm laser irradiation), EcN/CR, EcN/CR + L, EcN-IL-15/CR, and EcN-IL-15/CR + L. Bacteria (8 × 10^7^ CFU) were injected intravenously into tumor-bearing mice every 4 days. After 48 h of injection, mice in laser groups were irradiated on tumor sites by the 808 nm laser for 10 min (1.2 W cm^−2^). The therapeutic efficacy of the bacterial therapy was validated through three independent experiments, yielding similar results across these models. For anti-PD1 combinational therapy, mice were intraperitoneally injected with 250 μg anti-PD1 antibody (Bio X Cell, clone RMP1-14) every 3 days. The efficacy of the combinational therapy was commonly validated through repeated experiments across different tumor models (Colon26, 4T1-luc, and LLC), consistently yielding similar results.

#### Bacterial colonization

To detect bacterial distribution, Cy5-NHS dye was added to the bacterial solution and incubated at 37°C for 1 h to label the bacteria with Cy5. The solution was then washed three times with PBS until the supernatant became colorless. Colon26 tumor-bearing mice were intravenously injected with Cy5-labeled EcN-IL-15/CR or EcN (8 × 10^7^ CFU), or an equivalent dose of free dye. At designated time points, *in vivo* imaging was performed using an IVIS instrument (PerkinElmer, USA). At 96 h post-injection, tumors and major organs were dissected for imaging. To assess viable bacteria in tissues, the tissues were excised, homogenized, and diluted with PBS to appropriate concentrations. The bacterial solutions were then plated onto LB solid agar plates containing chloramphenicol and cultured at 37°C overnight. To investigate the location of EcN-IL-15/CR in tumors, tumor tissues from different regions were collected 120 h post-injection. The tissues were homogenized for colony counting. The remaining tissues were fixed with 2.5% glutaraldehyde and 2% paraformaldehyde at 4°C overnight, followed by three washes in PBS. The samples were then sent to the Southern Medical University for further processing and transmission electron microscopy (TEM) imaging.

#### CR detection

For the detection of CR content, Colon26 tumor-bearing mice were intravenously injected with EcN-IL-15/CR (8 × 10^7^ CFU). Tissues were collected 48 h post-injection. Tissues were rinsed, weighed, homogenized in RIPA lysis buffer on ice for 30 min, and centrifuged at 10,000 × g for 15 min at 4°C to obtain the supernatants. CR content in tissue extracts was measured using a fluorescence spectrophotometer.

#### Cytokine analysis

For the detection of IL-15 levels, Colon26 tumor-bearing mice were intravenously injected with EcN/CR or EcN-IL-15/CR (8 × 10^7^ CFU). Tissues and blood were collected 48 or 96 h post-injection. Tissues were weighed, homogenized in RIPA lysis buffer on ice for 30 min, and centrifuged at 10,000 × g for 15 min at 4°C to obtain the supernatants. Serum was prepared by allowing blood samples to clot at 4°C overnight, followed by centrifugation at 2,000 × g for 15 min at 4°C. IL-15 levels in tissues and serum were quantified using an ELISA kit according to the manufacturer’s instructions. For the detection of IFN-γ and IL-6 in serum, MC38 tumor-bearing mice were treated according to the experimental schedule and euthanized on day 9. Serum preparation was performed as described above, and cytokine levels were measured using ELISA kits following the manufacturer’s instructions.

#### Immune phenotyping analysis by flow cytometry

For tumor-infiltrating immune cell analysis, the tumor-bearing mice were treated as scheduled and euthanized on day 9. Tumors were harvested, mechanically minced, and then digested (tissue enzymatic digestion kit, RWD Life Science Co., LTD, America) in an incubator for 45 min at 37°C. Samples were filtered, washed, lysed in ACK buffer, and counted for flow cytometry analysis. 100 μL of single-cell suspension (∼2 × 10^6^ cells) was incubated with anti-mouse CD16/CD32 antibody (Bioxcell, clone 2.4G2, BE0307) according to the manufacturer’s specifications, and then incubated with specific panels of the following fluorochrome-labeled antibodies at 4°C for 30 min: CD45-PECF594 (BD Biosciences, 562420), CD3-FITC (BD Biosciences, 553062), CD4-APC (Biolegend, 100412), CD8a-APC-Cy7 (BD Biosciences, 557654), CD11b-APC-Cy7 (Biolegend, 101226), F4/80-APC (Biolegend, 123116), NKp46-FITC (Biolegend, 137606). For splenic and lymph node immune cell analysis, a similar procedure was followed, with the exception that tissue digestion was excluded. For memory T cell analysis, spleens were harvested, minced, and filtered through 70-μm cell strainers. Samples were lysed in ACK buffer and counted. 100 μL of single-cell suspension (∼2 × 10^6^ cells) was incubated with anti-mouse CD16/CD32 antibody, and then incubated with the following fluorochrome-labeled antibodies at 4°C for 30 min: CD45-BV570 (Biolegend, 103136), CD11b-APC-Cy7 (Biolegend, 101226), CD3-FITC (BD Biosciences, 553062), CD4-APC (Biolegend, 100412), CD8a-PE (Biolegend, 100708), CD62L-BV421 (Biolegend, 104435), CD44-AF700 (Biolegend, 103026). After washing, the antibody-stained cells were further stained with ViaDye Red to identify dead cells. Finally, the cells were detected using Cytek Aurora spectral flow cytometry and analyzed by Flowjo software (TreeStar, 10.6.2).

#### Splenocyte adoptive transfer

Spleens from mice that were treated with EcN/IL-15 + L or PBS were mechanically disaggregated and filtered 70-μm cell strainers. Red blood cells were lysed by ACK buffer and counted. 10 million splenocytes were intravenously injected into the tail vein of naive mice. One day later, Colon26 (1 × 10^6^ cells/100 μL) cells were implanted into the right flank of transferred mice. The day of tumor inoculations is defined as day 0. This experiment was performed once.

#### Immunofluorescent staining

The as-prepared tumor slides or cells were fixed by ice-cold acetone, methanol, or 4% paraformaldehyde, and blocked with TBST buffer containing 5% goat serum before antibody incubation. The primary antibodies involved include CD3 (Abcam, ab5690), CD8 (BD Biosciences, 550281), CD49b (BD Biosciences, 553855), HA (Cell Signaling Technology, 3724S), E.coli (Abcam, ab137967), CD11c (BD Biosciences, 550283), CALR (Abcam, ab92516), and HMGB1 (Abcam, ab18256). Fluorescence images were acquired using confocal laser scanning microscopy (Leica SP8, Germany).

#### RNA sequencing

Colon26 Tumors were harvested on day 9 from mice after two doses of bacteria treatment with irradiation. The samples were shipped to Sango Biotech company (Shanghai, China) for RNA extraction and transcriptome sequencing. Samples with sufficient quality were used for mRNA library construction and then sequenced on an Illumina NovaSeq platform (Novogene). Differential gene analysis was performed using DESeq2 package, identifying genes with a *p*-value of less than 0.05 and |log2(fold change)| > 1 as significantly differentially expressed. For KEGG pathway enrichment analysis, we employed the clusterProfiler package in R. Genes identified as differentially expressed were mapped to KEGG pathways using the “enrich KEGG” function. Multiple testing correction was applied using the Benjamini-Hochberg procedure.

#### Biosafety assay

The Colon26-bearing mice were treated as scheduled in Figure 4A and sacrificed on day 9. Blood samples were collected for blood biochemical tests. The major organs were collected for H&E staining. For the time-course biosafety study, Colon26-bearing mice were intravenously injected with EcN-IL-15/CR (8 × 10^7^ CFU), followed by 808 nm laser irradiation (L) 48 h post-injection. Body temperature was monitored daily throughout the study. Mice were euthanized at 24, 48, and 96 h post-injection, with blood samples collected for biochemical analysis and organs harvested for H&E staining.

### Quantification and statistical analysis

Data are presented as means ± SEM, unless otherwise specified. Statistical analyses were performed using GraphPad Prism version 8. The statistical significance was analyzed using either two-tailed Student’s t test, one-way analysis of variance (ANOVA), or two-way ANOVA as indicated in figure legends. Survival analysis was performed by a log rank test. Differences were considered significant if *p* < 0.05 (∗*p* < 0.05, ∗∗*p* < 0.01, ∗∗∗*p* < 0.001, and ∗∗∗∗*p* < 0.0001).

## Supplemental information

Document S1. Figures S1–S20, Table S1, and Scheme S1

Document S2. Article plus supplemental information
