# Supplementary material for: Probiotic-mediated tumor microenvironment reprogramming with protease-sensitive interleukin-15 and photothermal therapy
Source: Cell Rep Med. 2025 Jun 17;6(6):102191. doi: 10.1016/j.xcrm.2025.102191 (PMC12208336; doi:10.1016/j.xcrm.2025.102191)
Supplement: Document S1. Figures S1–S20, Table S1, and Scheme S1 [file mmc1.pdf]

Cell Reports Medicine, Volume *6*

Supplemental information

Probiotic-mediated tumor microenvironment reprogramming with protease-sensitive interleukin-15 and photothermal therapy

Huifang Wang, Liuhai Zheng, Chuanbin Yang, Lin Jia, Runhua Zhou, Hongda Liu, Yafang Dong, Xiaolong Xu, Guangwei Shi, Jialu Yang, Yang Li, Haitao Yuan, Jinpeng Cen, Guiming Zhang, Le Yu, Tianqi Guo, Haibo Jiang, Yawei Liu, Xijun Wang, Zhijie Li, and Jigang Wang

Supplementary Figures

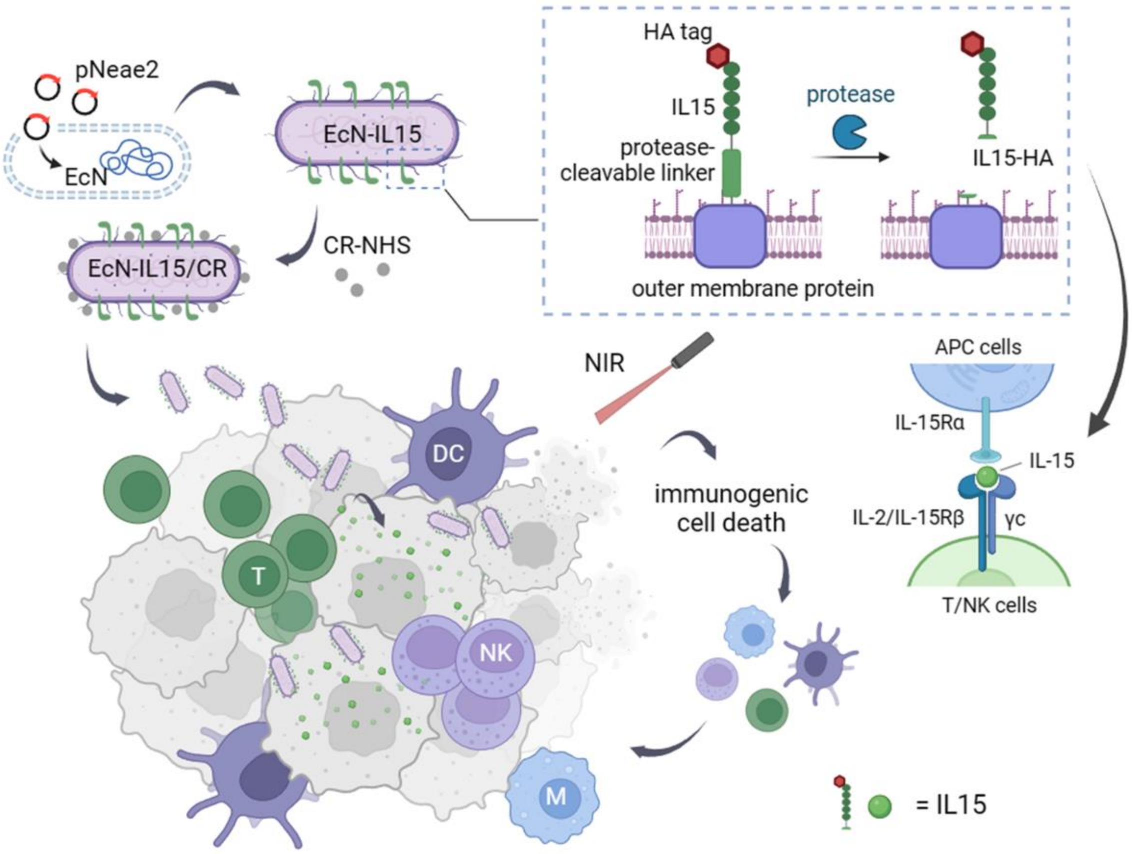

Scheme S1. Reactivating antitumor immunity with protease-sensitive interleukin-15 and photothermal therapy mediated by bioengineered probiotics. Related to Figure 1.

A recombinant plasmid (pNeae2) encoding conditionally-released IL-15 protein is introduced into E. coli Nissle 1917 (EcN) to produce EcN-IL-15, with the goal of releasing IL-15 within a protease-enriched tumor microenvironment (TME). Croconium dye (CR), a photothermal agent, is further conjugated with EcN-IL-15 to induce immunogenic cell death (ICD) within tumor sites. The resulting EcN-IL-15/CR bacteria are able to home to tumors, leveraging their inherent tropism for hypoxic regions. CR-mediated photothermal therapy promotes the infiltration of T cells, NK cells, DCs, and macrophages, while bacterium-derived IL-15 boosts the proliferation and survival of T cells and NK cells.

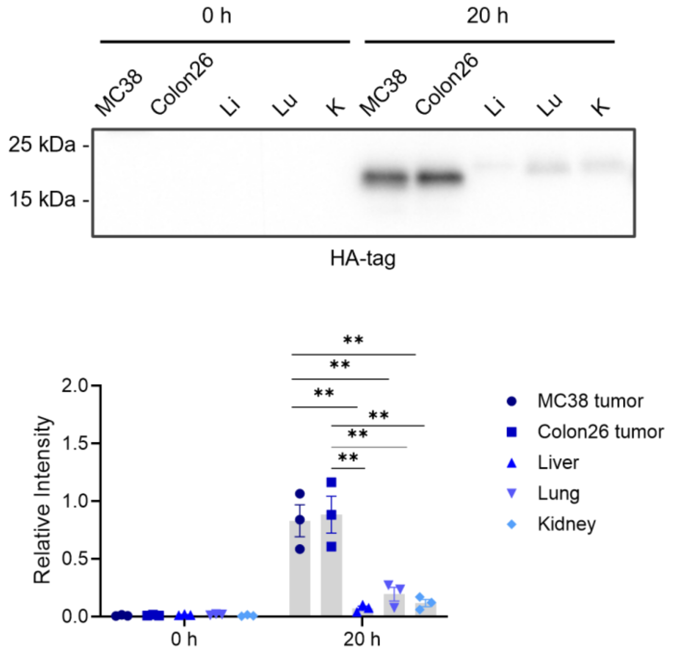

Figure S1. EcN-IL-15 cleavage by mouse tumors. Related to Figure 1.

EcN-IL-15 bacteria (2 × 10^8 CFU) were incubated with either tumor or other tissue homogenates (2 mg mL^{-1}) for 20 hours at 37 °C. Then the bacteria were pelleted through centrifugation. The cleavage of IL-15-HA in the supernatant of the reaction mixture was analyzed by Western blotting; n = 3 biological replicates. Li, liver; Lu, lung; K, kidney. Data are presented as mean ± SEM. *P*-values were calculated using one-way ANOVA with Tukey's multiple comparisons. $^{**}P < 0.01$.

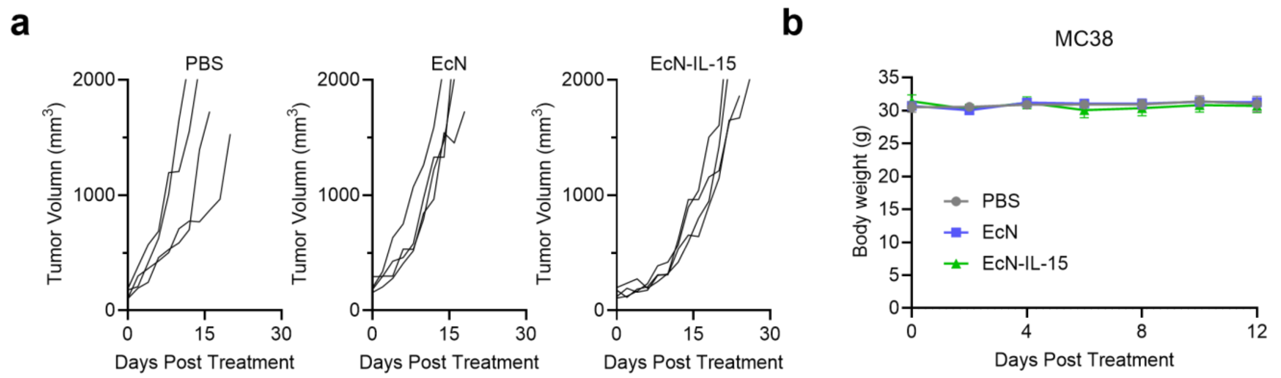

Figure S2. EcN-IL-15 delayed MC38 tumor progression *in vivo*. Related to Figure 1.

(**a**) Tumor growth curves of individual mice, corresponding to Figure 1i.

(**b**) Preliminary safety evaluation of EcN-IL-15. Body weight changes of MC38-bearing mice that were intravenously treated with PBS, EcN, or EcN-IL-15 (2 injections in total), respectively; n = 4 biological replicates.

Data are presented as mean ± SEM.

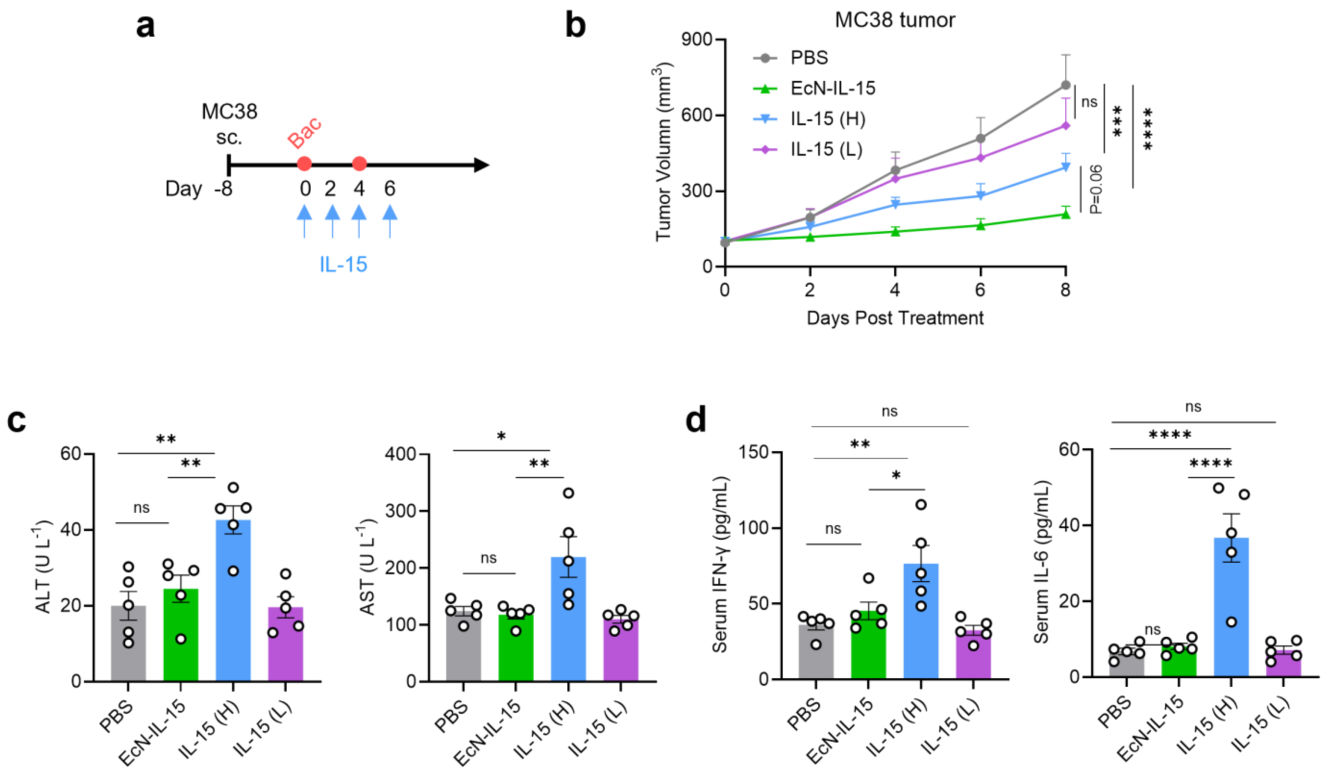

Figure S3. EcN-IL-15 bacteria show reduced toxicity compared to high-dose IL-15. Related to Figure 1.

(**a**) Treatment schedule in subcutaneous MC38 tumor model. MC38-bearing mice were treated intravenously with PBS, EcN-IL-15 (4×10^7 CFU, two injections in total), or with recombinant mouse IL-15 protein (four injections in total). High-dose IL-15 (H) was administered at 10 μg/mouse, while low-dose IL-15 (L) was given at 5 μg/mouse. Blood was collected on day 9 for analysis.

(**b**) MC38 tumor growth curves with different treatments; n = 5-6 biological replicates.

(**c**) Serum ALT and AST levels; n = 5 biological replicates.

(**d**) Serum IFN-γ and IL-6 levels measured by ELISA; n = 5 biological replicates.

Data are presented as means ± SEM. *P*-values were analyzed by two-way ANOVA with Holm-Sidak's multiple comparisons test (**b**), or one-way ANOVA with Tukey's multiple comparisons test **(c, d**). *$P < 0.05$, **$P < 0.01$, ***$P < 0.001$, ****$P < 0.0001$; ns, not significant.

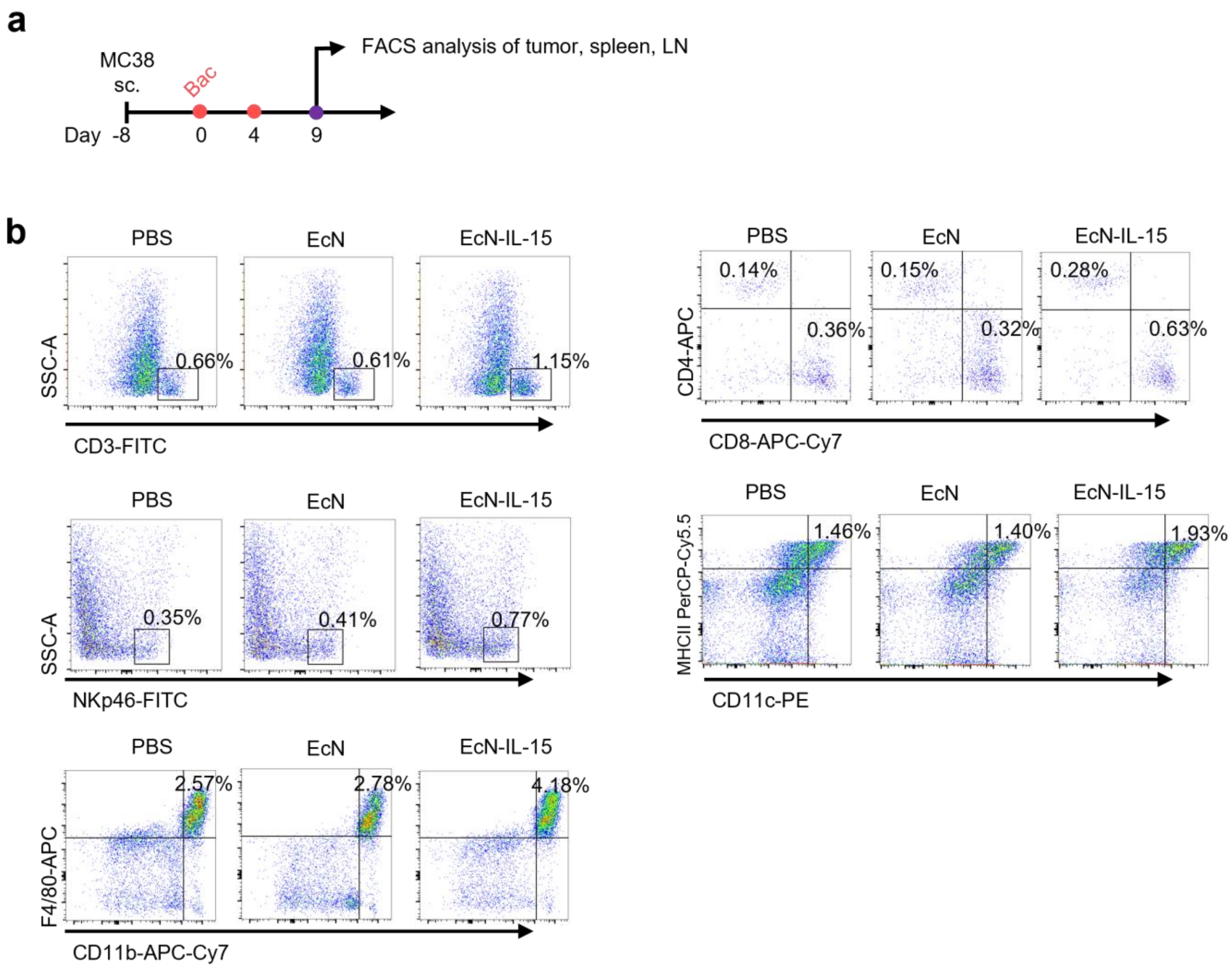

Figure S4. Evaluation of immune cell alterations in the tumor microenvironment following EcN-IL-15 bacteria therapy. Related to Figure 1.

(**a**) Treatment schedule of EcN-IL-15 in subcutaneous MC38 tumor. MC38-bearing mice were treated intravenously with PBS, EcN, or EcN-IL-15 (4×10^7 CFU, two injections in total) as indicated. Mice were sacrificed on day 9 for flow cytometry analysis; n = 4 biological replicates.

(**b**) Representative flow cytometric plots of $CD3^+$ T cells, $CD4^+$ T cells, $CD8^+$ T cells, NK cells, DCs, and macrophages, corresponding to Figures 1k.

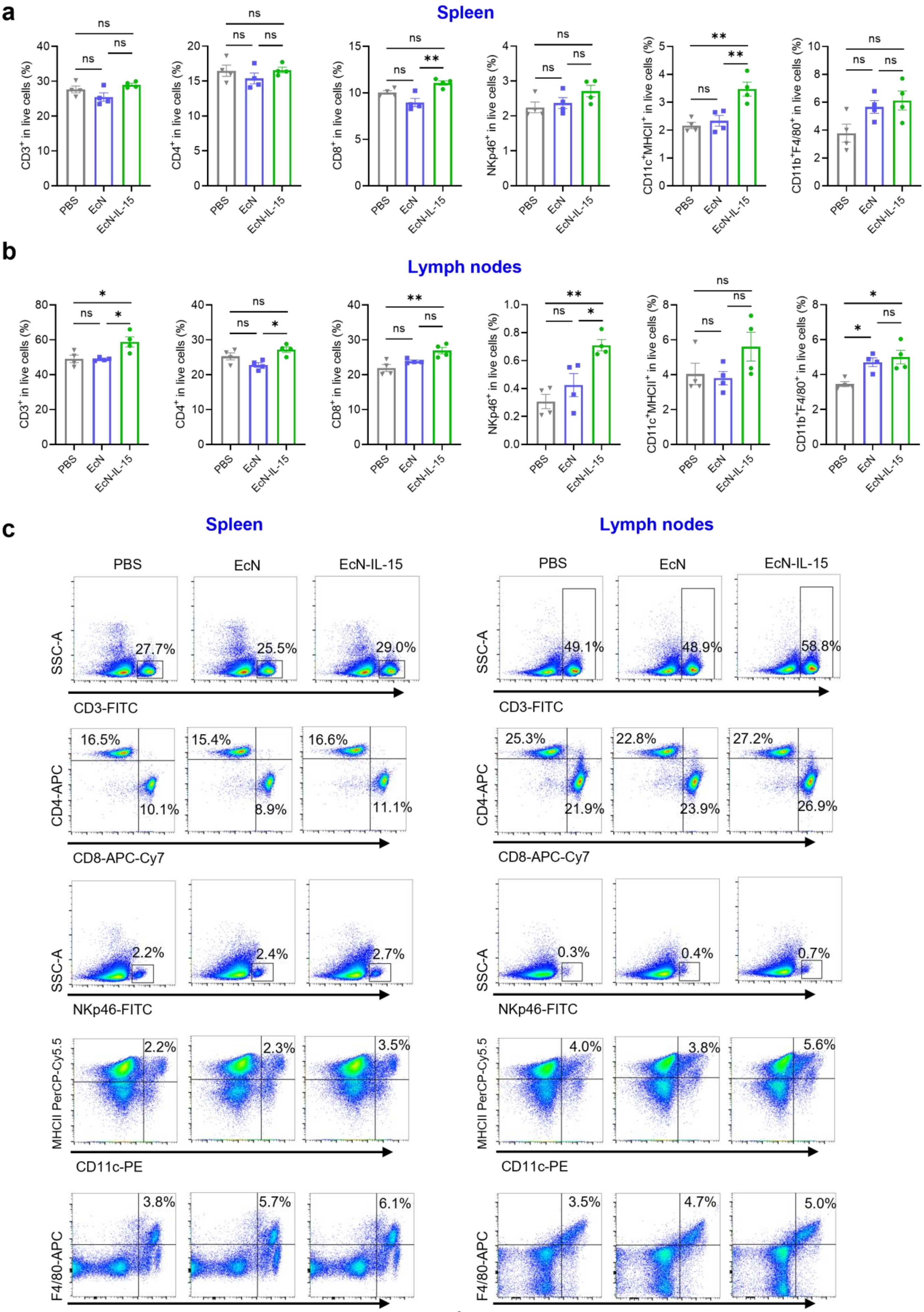
a
Spleen
CD3+ in live cells (%)
CD4+ in live cells (%)
CD8+ in live cells (%)
NKp46+ in live cells (%)
CD11c+MHCII+ in live cells (%)
CD11b+F4/80+ in live cells (%)
PBS
EcN
EcN-IL-15
ns
*
**
b
Lymph nodes
c
Spleen
Lymph nodes
PBS
EcN
EcN-IL-15
SSC-A
CD3-FITC
27.7%
25.5%
29.0%
49.1%
48.9%
58.8%
CD4-APC
CD8-APC-Cy7
16.5%
10.1%
15.4%
8.9%
16.6%
11.1%
25.3%
21.9%
22.8%
23.9%
27.2%
26.9%
NKp46-FITC
2.2%
2.4%
2.7%
0.3%
0.4%
0.7%
MHCII PerCP-Cy5.5
CD11c-PE
2.2%
2.3%
3.5%
4.0%
3.8%
5.6%
F4/80-APC
CD11b-APC-Cy7
3.8%
5.7%
6.1%
3.5%
4.7%
5.0%

Figure S5. Evaluation of immune cell alterations in the spleen and lymph nodes following EcN-IL-15 bacteria therapy. Related to Figure 1.

(a-b) Quantification of $CD3^+$ T cells ($CD45^+CD3^+$), $CD4^+$ T cells ($CD45^+CD3^+CD4^+$), $CD8^+$ T cells ($CD45^+CD3^+CD8^+$), NK cells ($CD45^+NKp46^+$), DCs ($CD45^+CD11c^+MHCII^+$), and macrophages ($CD45^+CD11b^+F4/80^+$) in the spleen (**a**) and lymph nodes (**b**), as determined by flow cytometry; n = 4 biological replicates.

(**c**) Representative flow cytometric plots of $CD3^+$ T cells, $CD4^+$ T cells, $CD8^+$ T cells, NK cells, DCs, and macrophages, corresponding to Figures S5a-b.

Data are presented as mean ± SEM. *P*-values were calculated using one-way ANOVA with Tukey's multiple comparisons test (**a-b**). $^*P < 0.05$, $^{**}P < 0.01$; ns, not significant.

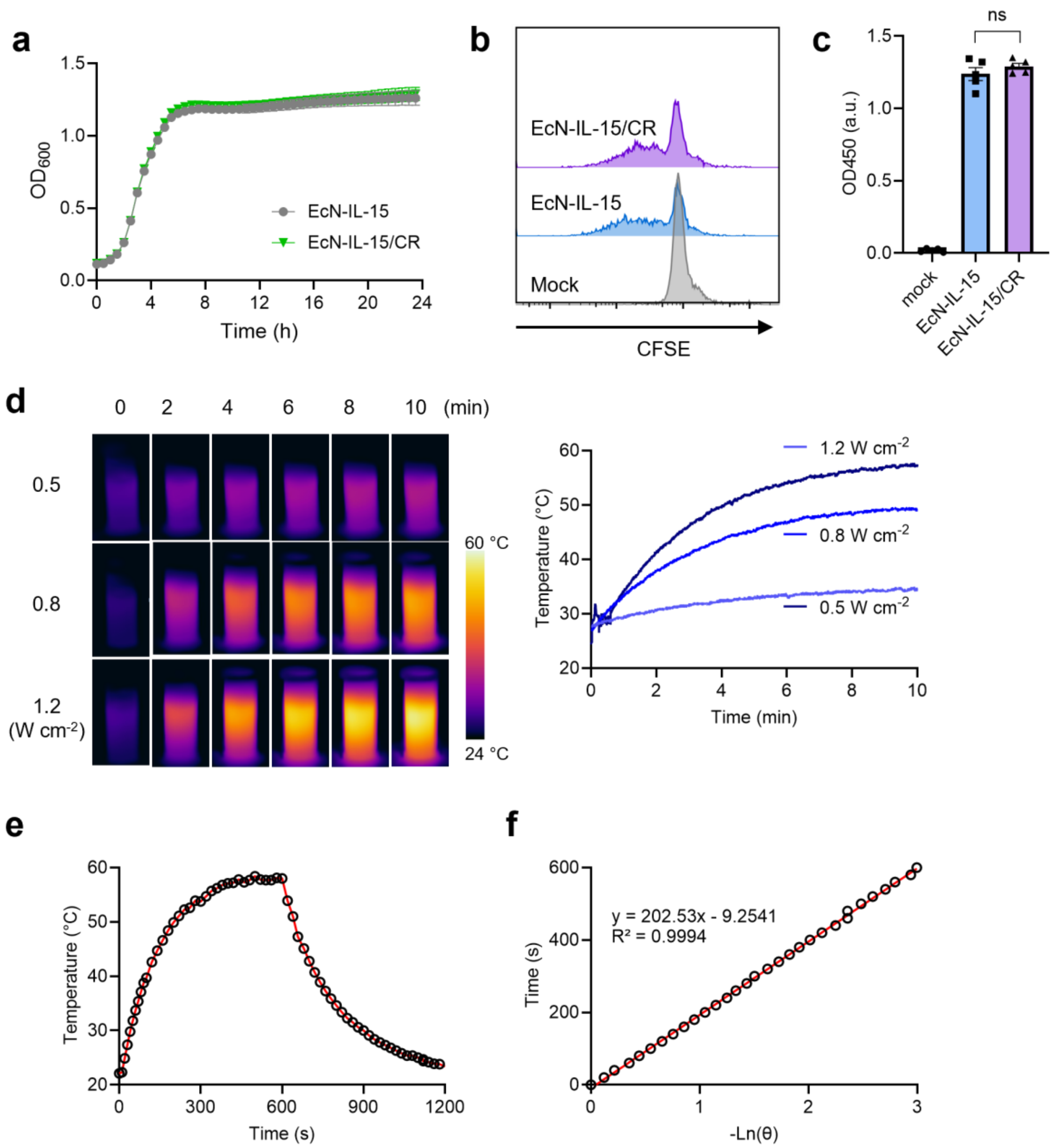

Figure S6. Preparation and characterization of EcN-IL-15/CR. Related to Figure 2.

(**a**) Growth curves of EcN-IL-15 before and after CR modification. Bacteria were incubated in an LB medium at 37 °C. Optical density at 600 nm (OD600) was recorded at 30-minute intervals using a microplate reader; n = 3 biological replicates. Data are presented as means ± SD.

(**b-c**) EcN-IL-15/CR bacteria stimulate splenocyte proliferation similarly to EcN-IL-15. CFSE-labeled mouse splenocytes were stimulated for 72 hours with the supernatant from MMP2 cleavage of EcN-IL-15 or EcN-IL-15/CR, followed by flow cytometry analysis (**b**) and CCK8 assay (**c**); n = 3 biological replicates.

(**d**) Representative thermal images and temperature variations of EcN/CR (4×10^8 CFU mL^{-1}) after 808 nm laser irradiation at different power densities (0.5, 0.8, and 1.2 W cm^{-2}) for 10 minutes.

(**e-f**) Heating and cooling profiles of CR-modified bacteria under 808 nm laser irradiation. Temperature-time curves of CR-modified bacteria under 808 nm laser irradiation. The bacterial solution (4×10^8 CFU mL^{-1}) was irradiated with an 808 nm laser at a power density of 1.2 W cm^{-2} for 10 minutes (heating phase), followed by a cooling phase after laser cessation (**e**).

Temperature variations were monitored in real-time using a thermal camera. Linear fit of cooling time (t) vs. negative natural logarithm of driving force temperature [-ln(θ)] obtained from the cooling phase. The time constant was determined to be 202.53 s based on the fitted data (**f**).

Data are presented as means ± SEM, unless otherwise specified. *P*-values were calculated using one-way ANOVA with Tukey's multiple comparisons test (**c**). ns, not significant.

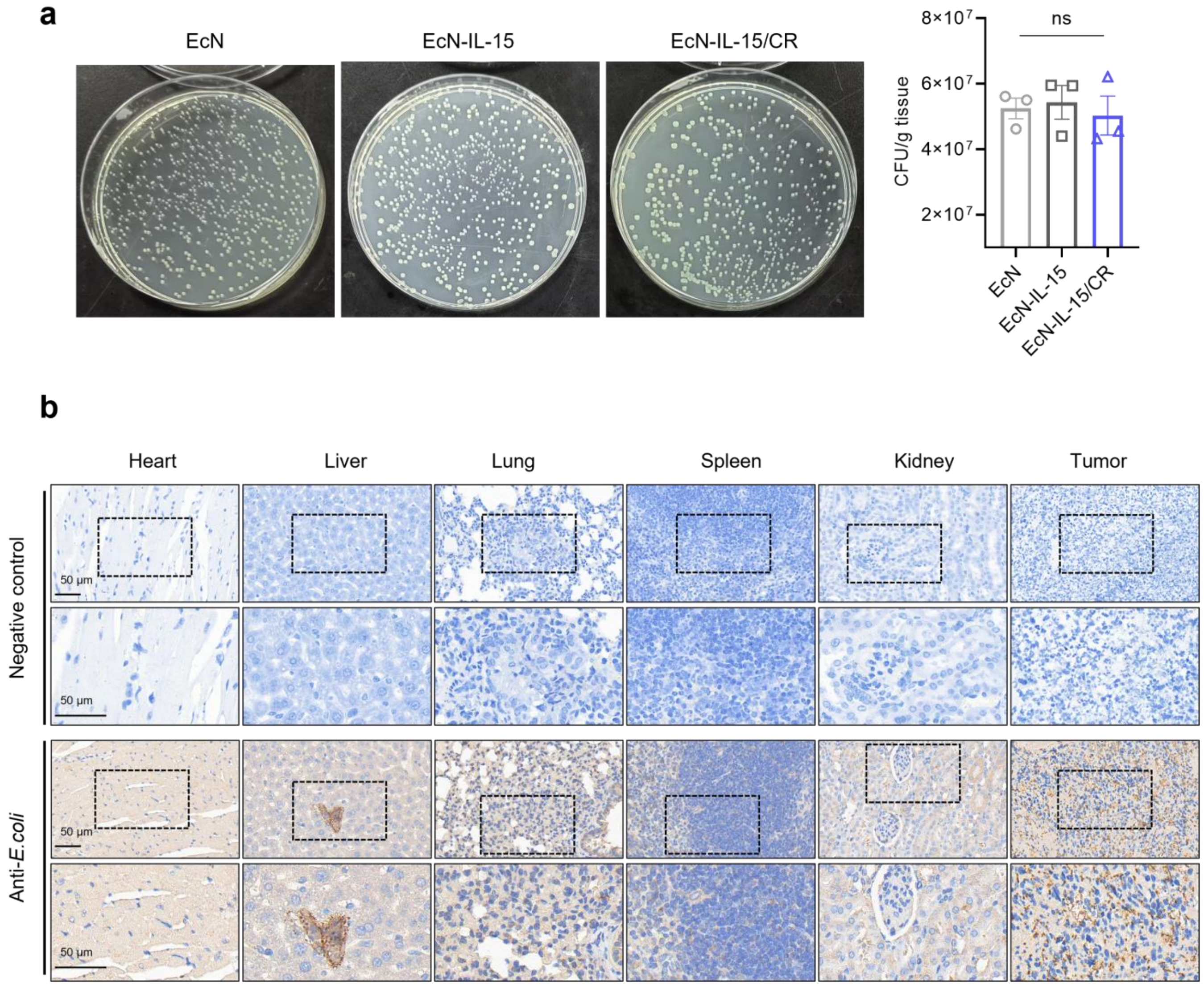

Figure S7. *In vivo* tumor homing of EcN-IL-15/CR. Related to Figure 3.

(**a**) Representative photographs and quantification of bacterial colonization in tumors treated with different engineered bacteria. Colon26-bearing mice were intravenously injected with EcN, EcN-IL-15, or EcN-IL-15/CR (8×10^7 CFU). Tumors were harvested for analysis at 48 hours post-injection. n = 3 biological replicates.

(**b**) Representative images of immunohistochemical staining for *E. coli*, depicting the bacterial distribution in major organs and tumors. Colon26 tumor-bearing mice were intravenously injected with EcN-IL-15/CR (8×10^7 CFU), and tissues were harvested for analysis 48 hours post-injection. Slides were stained using an anti-*E. coli* antibody (Abcam, ab137967, 1:200). Representative images of three mice are shown, with ~5 fields of view per sample. High-magnification images correspond to the areas marked by dotted black lines. Scale bars, 50 μm. Negative control staining means that no primary antibodies were applied for the staining.

Data are presented as mean ± SEM. *P*-values were calculated using one-way ANOVA with Tukey's multiple comparisons test (**a**). ns, not significant.

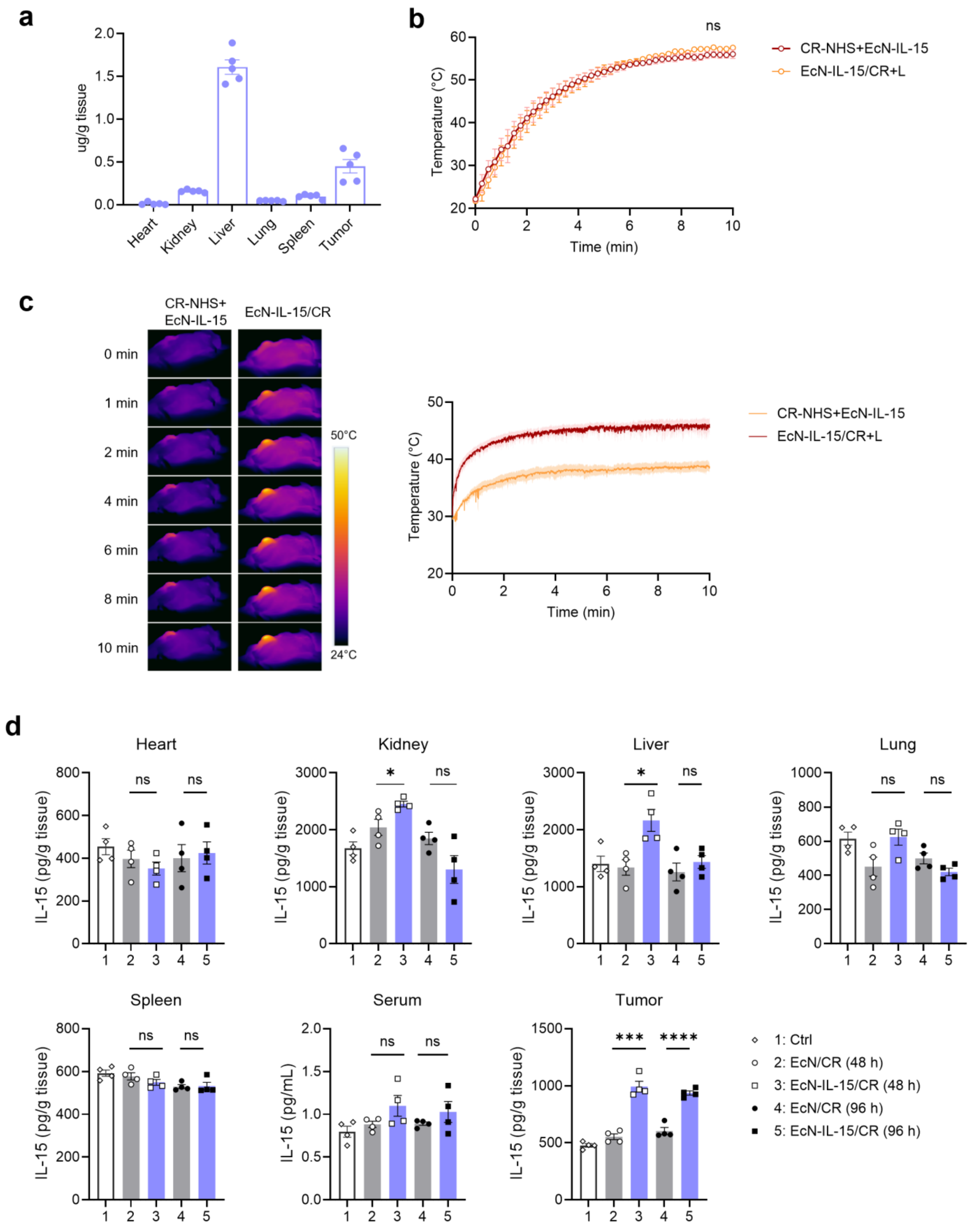

Figure S8. *In vivo* CR biodistribution and IL-15 levels in tumor-bearing mice. Related to Figure 3.

(a) Quantification of CR biodistribution of EcN-IL-15/CR**.** Colon26 tumor-bearing mice were intravenously injected with EcN-IL-15/CR (8 × 10^7 CFU) and sacrificed 48 hours post-injection. CR content in tissue extracts was measured using a fluorescence spectrophotometer. n = 5 biological replicates.

(b-c) Comparison of thermal effects between CR-modified bacteria and free CR dye. EcN-IL-15/CR solution (4×10^8 CFU mL^{-1}) and a mixture solution containing EcN-IL-15 and CR-NHS (equivalent concentrations of CR-NHS) were exposed to 808 nm laser irradiation (1.2 W cm^{-2}) for 10 minutes (n = 3) (**b**). For in vivo experiments, mice were intravenously injected with the EcN-IL-15/CR or CR-NHS plus EcN-IL-15. In the CR-NHS plus EcN-IL-15 group, CR-NHS dye was administered first, followed by EcN-IL-15 bacteria (8×10^7 CFU) 2 hours later. Laser irradiation (808 nm, 1.2 W cm^{-2}, 10 minutes) was performed 48 hours post-injection (n = 3) (**c**). Thermal images were captured using a thermal camera to visualize and quantify the thermal effects.

(d) Tissue and serum IL-15 levels in tumor-bearing mice after treatment with engineered bacteria. Colon26 tumor-bearing mice were intravenously injected with EcN/CR or EcN-IL-15/CR (8×10^7 CFU). Tissues and serum were collected at 48 hours or 96 hours post-injection. IL-15 levels were quantified by ELISA. n = 4 biological replicates. 1, Ctrl; 2, EcN/CR (48 h); 3, EcN-IL-15/CR (48 h); 4, EcN/CR (96 h); 5, EcN-IL-15/CR (96 h).

Data are presented as mean ± SEM. *P*-values were calculated using two-way ANOVA with Sidak's multiple comparisons test (**b**), or one-way ANOVA with Tukey's multiple comparisons test (**d**). $^*P < 0.05$, $^{***}P < 0.001$, $^{****}P < 0.0001$; ns, not significant.

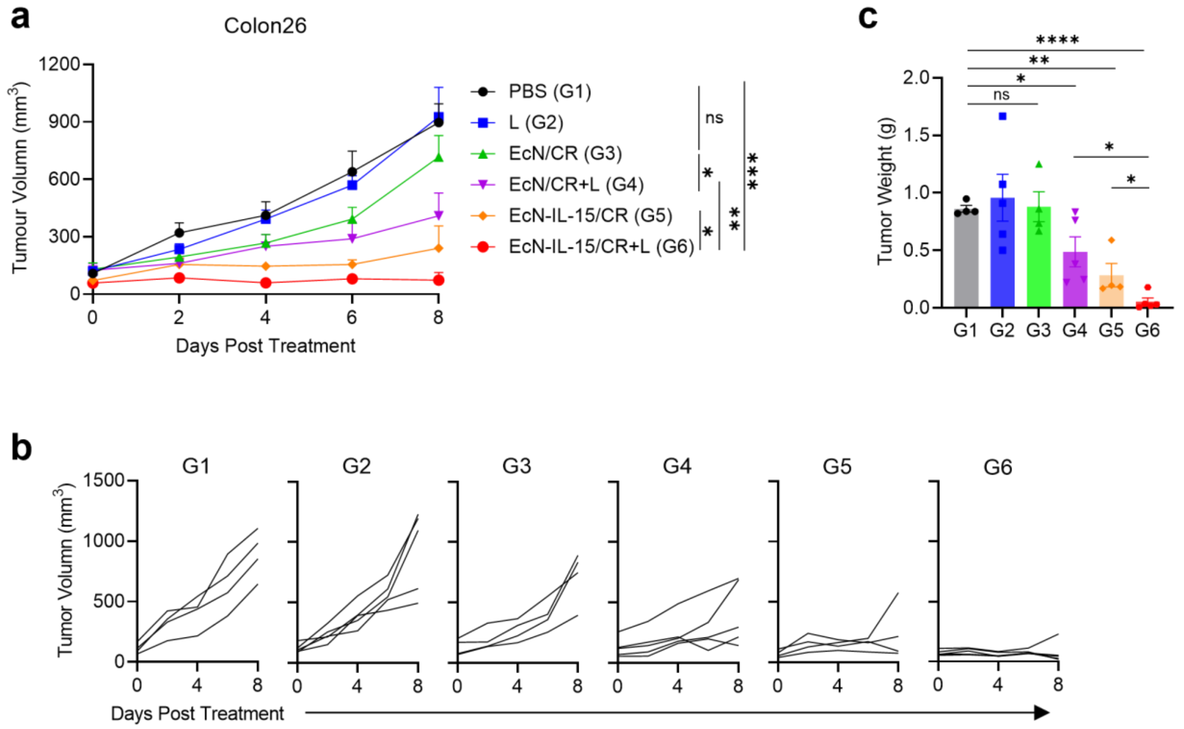

Figure S9. Antitumor effect of photothermal EcN-IL-15/CR on Colon26 colorectal cancer. Related to Figure 4.

(**a-b**) Colon26 tumor growth curves; Colon26-bearing mice were intravenously injected with PBS, EcN/CR, or EcN-IL-15/CR (8×10^7 CFU), followed by 808 nm laser irradiation (L) at 48 h post-injection, as shown in Figure 4a; n = 4–5 biological replicates.

(**c**) Masses of dissected tumors on day 9; n = 4–5 biological replicates.

G1, PBS; G2, laser alone; G3, EcN/CR; G4, EcN/CR+laser; G5, EcN-IL-15/CR; G6, EcN-IL-15/CR+laser. Data are presented as means ± SEM. *P*-values were analyzed by two-way ANOVA with Tukey's multiple comparisons test (**a**)**,** a two-way ANOVA with Sidak's multiple comparisons test between G5 and G6) (**a**), or two-tailed unpaired Student's t-test (**c**). *P < 0.05, **P < 0.01, ***P < 0.001, ****P < 0.0001; ns, not significant.

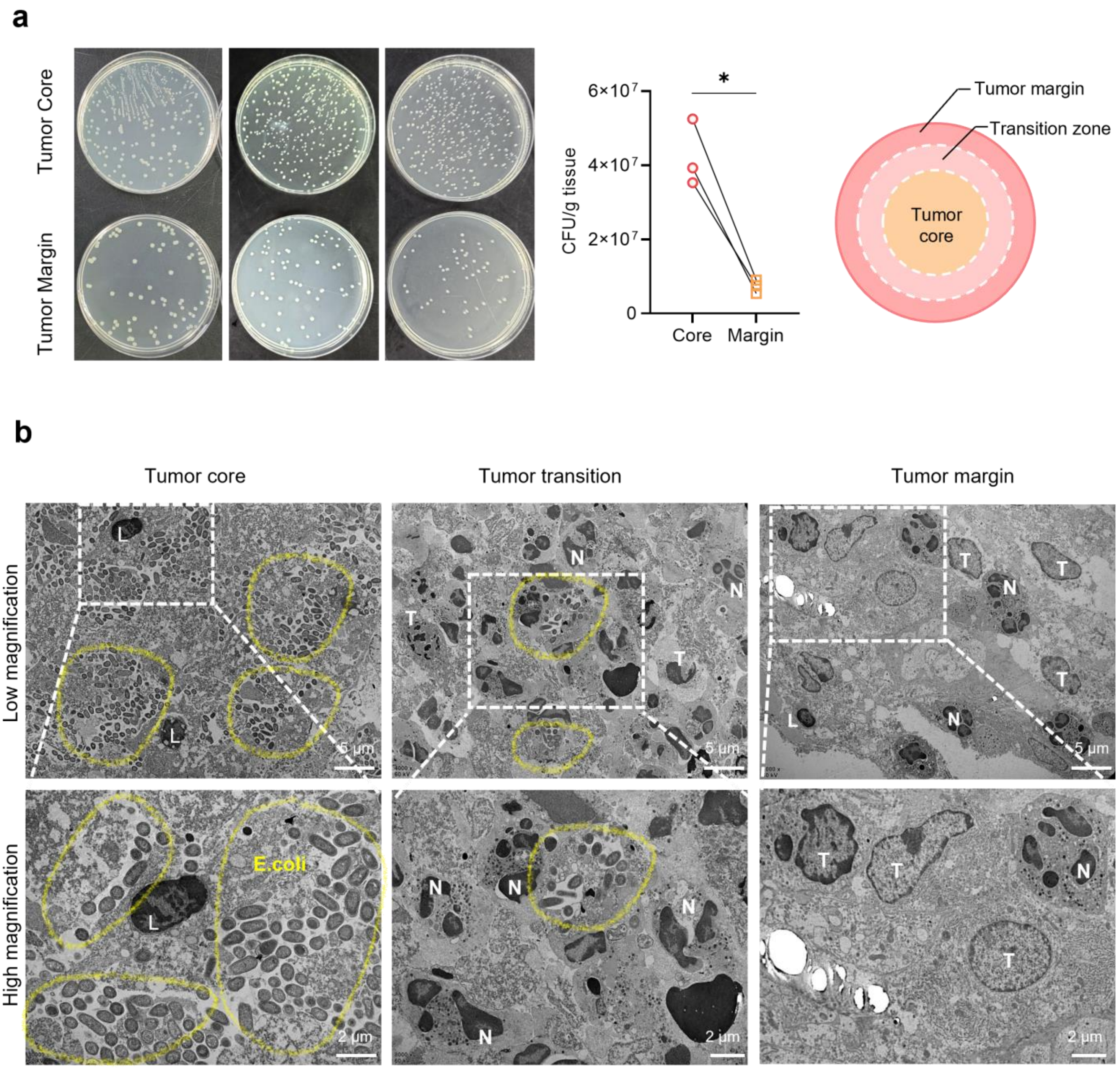

Figure S10. Bacteria preferentially accumulate in the tumor core regions. Related to Figure 4.

(a) Distribution of bacterial colonies in tumor core and margin areas. Colon26-bearing mice were intravenously injected with EcN-IL-15/CR (8 × 10^7 CFU), followed by 808 nm laser irradiation 48 hours after injection. Tumor tissues from distinct regions were collected 120 hours post-injection. n = 3 biological replicates.

(b) Transmission electron microscopy of tumor tissue showing bacterial colonization in distinct regions. Yellow circles indicate the presence of *E. coli* within the tumor tissue (n = 3). These bacteria are characterized by a dark, uniform electron density. Scale bars: 5 μm (low magnification), 2 μm (high magnification). L, N, and T indicate lymphocytes, neutrophils, and tumor cells, respectively.

P-values were calculated using two-tailed paired Student's t-test (**a**). $^{*}P < 0.05$.

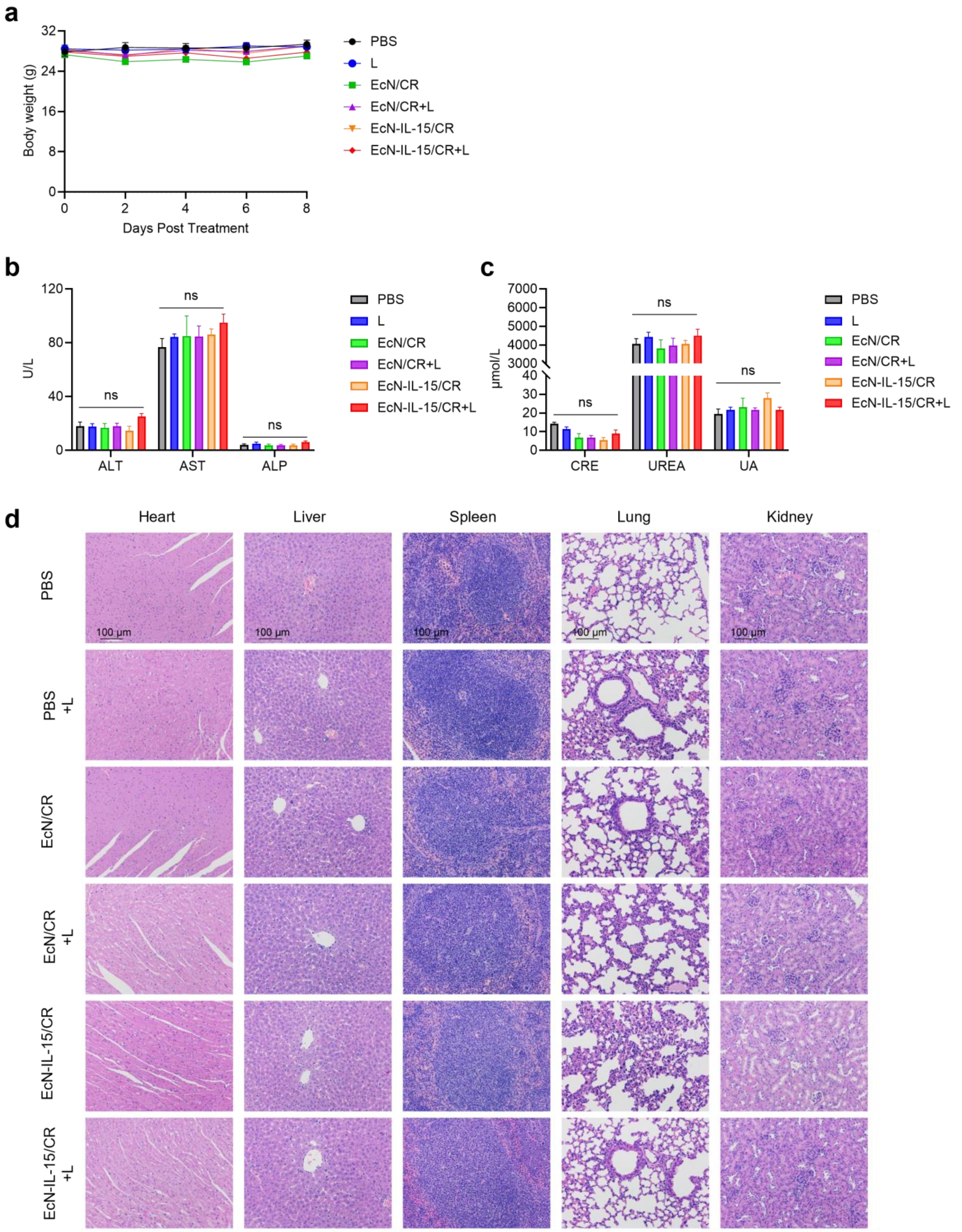

Figure S11. *In vivo* safety evaluation. Related to Figure 4. Colon26-bearing mice were intravenously injected with PBS, EcN/CR, or EcN-IL-15/CR (8 × 10^7 CFU) on day 0 and day 4, followed by 808 nm laser irradiation (L) 48 hours post-injection, as described in Figure 4a. On day 9, blood samples were collected for biochemical analysis, and organs were

harvested for pathological analysis.

(**a**) Body weight changes of Colon26-bearing mice after various treatments. n = 4-5 biological replicates.

(**b**) Liver function evaluation in Colon26-bearing mice. ALT, alanine aminotransferase; AST, aspartate aminotransferase; ALP, alkaline phosphatase. n = 4-5 biological replicates.

(**c**) Kidney function evaluation in Colon26-bearing mice. CRE, creatinine; UA, uric acid. n = 4-5 biological replicates.

(**d**) Representative H&E staining of major organs harvested from the Colon26-bearing mice on day 9. Representative images of three mice are shown. Scale bars, 100 μm.

Data are presented as means ± SEM. *P*-values were analyzed by two-way ANOVA with Tukey's multiple comparisons test. ns, not significant.

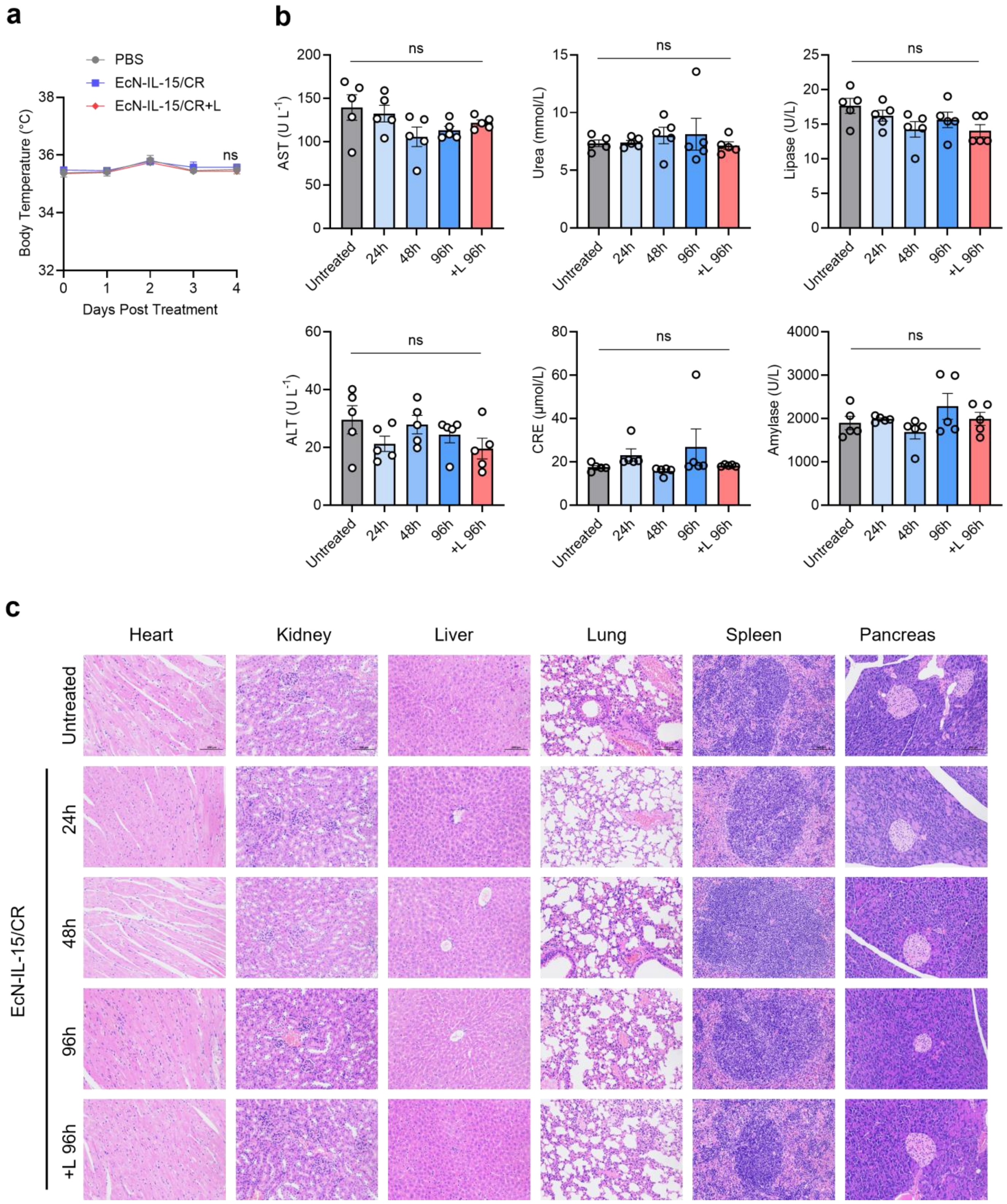

Figure S12. Time-course safety evaluation in tumor-bearing mice following bacterial therapy. Related to Figure 4. Colon26-bearing mice were intravenously injected with EcN-IL-15/CR (8×10^7 CFU), followed by 808 nm laser irradiation (L) at 48 hours post-injection. Body temperature was monitored daily throughout the study. Mice were sacrificed at 24-, 48-, and 96-hours post-injection, with blood samples collected for biochemical analysis and organs harvested for pathological evaluation.

(**a**) No significant changes in body temperature were observed following bacterial therapy. n = 5 biological replicates.

(**b-c**) Bacterial therapy did not cause significant liver, kidney, or pancreatic toxicity in tumor-bearing mice. Serum liver function markers (AST, ALT), kidney function markers (urea, CRE), and pancreatic injury markers (lipase, amylase) showed no significant differences between treated and untreated mice (**b**). n = 5 biological replicates. ALT, alanine aminotransferase; AST, aspartate aminotransferase; CRE, creatinine. Histopathological analysis of liver, kidney, and pancreas tissues showed no visible signs of damage (**c**). Representative images of three mice are shown. Scale bars, 100 µm.

Data are presented as means ± SEM. *P*-values were analyzed by two-way ANOVA with Tukey's multiple comparisons test (**a**), or one-way ANOVA with Tukey's multiple comparisons test (**b**). ns, not significant.

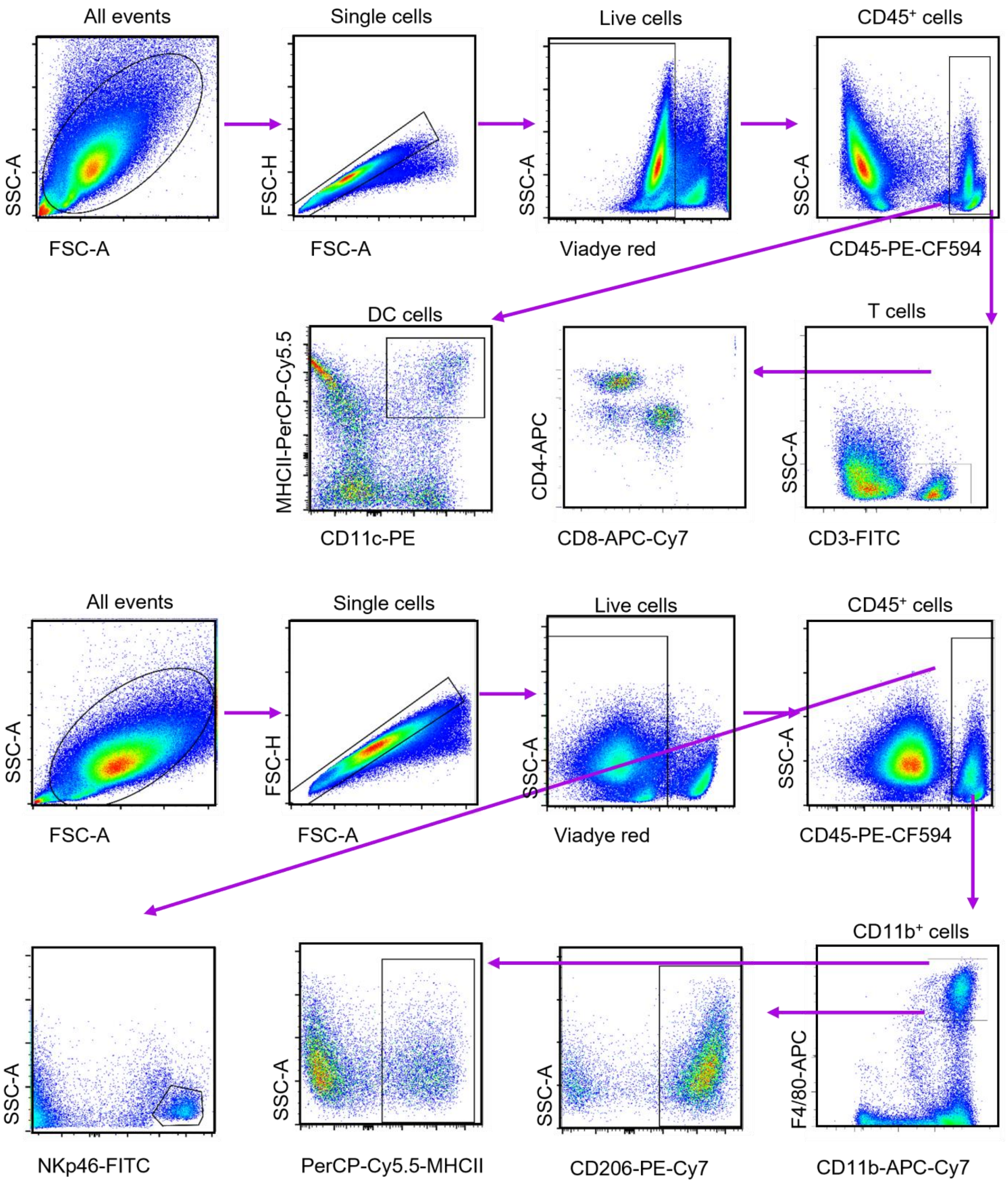

Figure S13. Gating strategy for flow cytometric analysis of immune cells in tumors on day 9. Related to Figures 1 and 5.

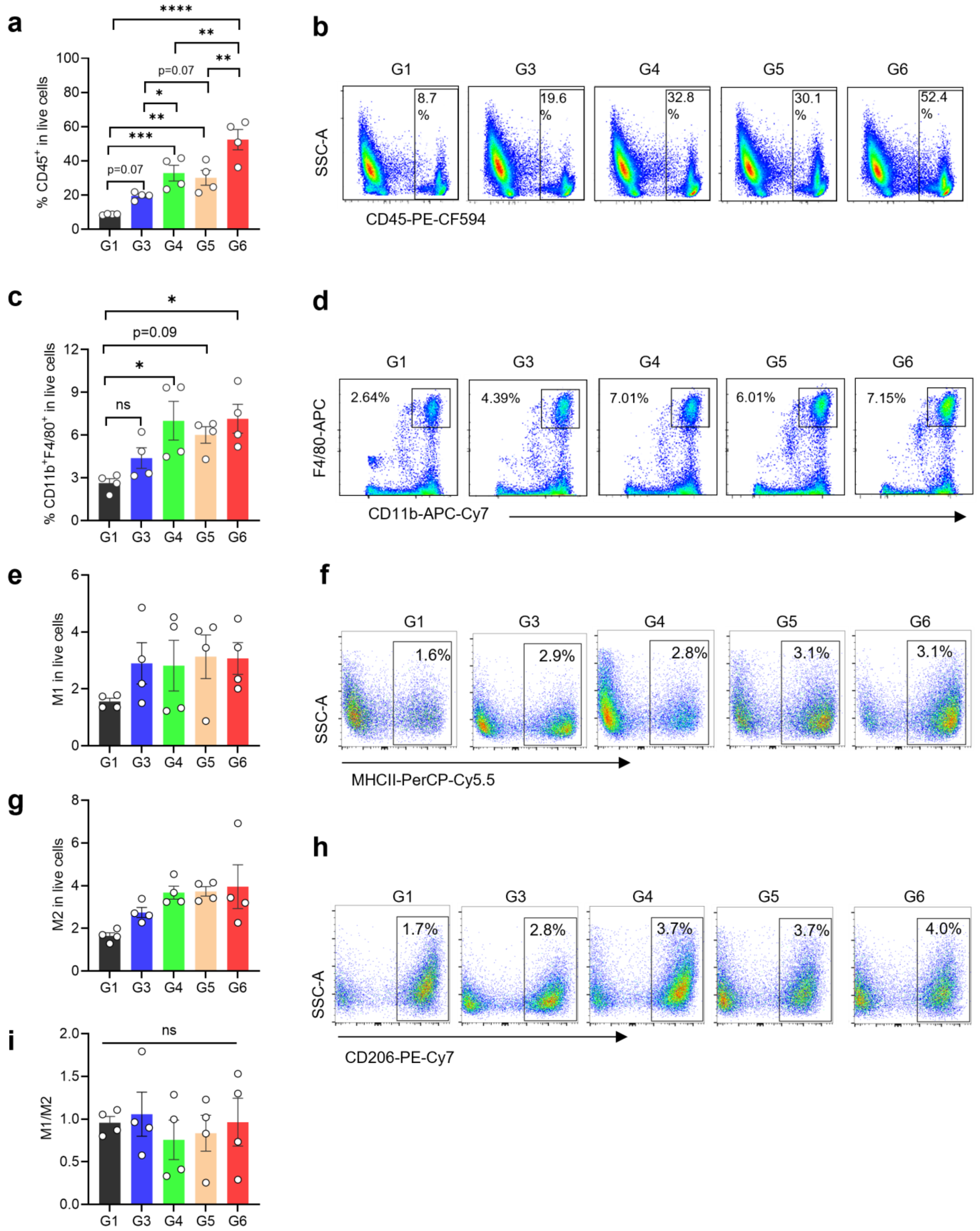

Figure S14. Infiltration of immune cells in Colon26 tumors after various treatments. Related to Figure 5.

(**a-b**) Flow cytometric analysis and quantification of leukocytes (CD45$^+$) in Colon26 tumors; n = 4 biological replicates.

(**c-d**) Flow cytometric analysis and quantification of macrophages (CD45$^+$CD11b$^+$F4/80$^+$) in Colon26 tumors; n = 4

biological replicates.

(**e-i**) Flow cytometric analysis and quantification of M1 ($CD45^+CD11b^+F4/80^+MHCII^+$) and M2 macrophages ($CD45^+CD11b^+F4/80^+CD206^+$) in Colon26 tumors; n = 4 biological replicates.

G1, PBS; G3, EcN/CR; G4, EcN/CR+Laser; G5, EcN-IL-15/CR; G6, EcN-IL-15/CR+Laser. Data are presented as means ± SEM. *P*-values were analyzed by one-way ANOVA with two-stage linear step-up procedure of Benjamini, Krieger and Yekutieli test to control the false discovery rate (**a**), or one-way ANOVA with Tukey's multiple comparisons test (**c, i**). *P < 0.05, **P < 0.01, ***P < 0.001, ****P < 0.0001; ns, not significant.

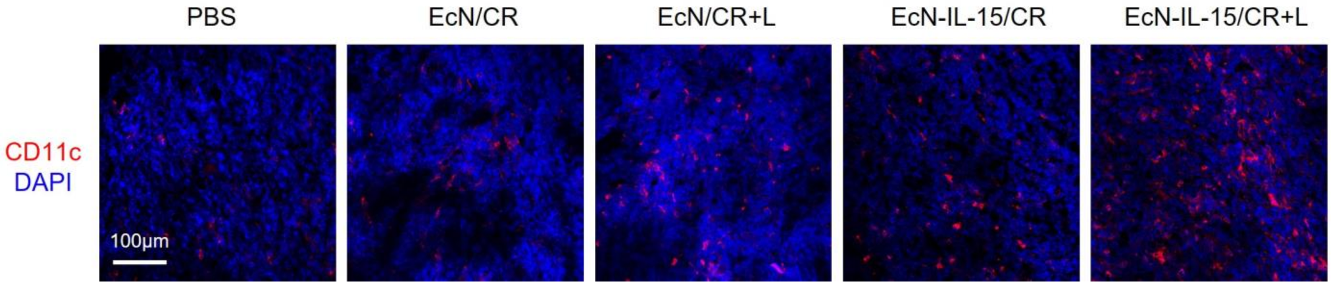

Figure S15. Infiltration of DCs in Colon26 tumors after various treatments. Related to Figure 5.

Immunostaining of CD11c (red) in Colon26 tumor sections. The cell nuclei were stained with DAPI (blue). The images are representative of three mice, with ~5 fields of view per sample. Scale bars, 100 μm.

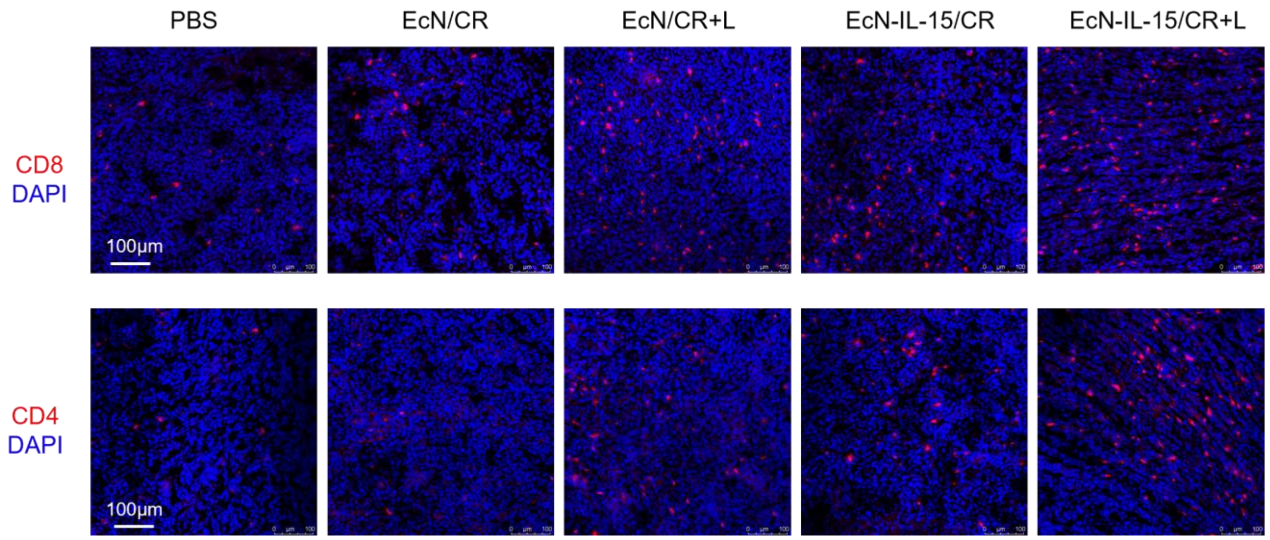

Figure S16. Infiltration of T cells in Colon26 tumors after various treatments. Related to Figure 5.

Infiltration of T cells in Colon26 tumors after various treatments. Immunostaining of CD8 (red) or CD4 (red) in Colon26 tumor sections. The cell nuclei were stained with DAPI (blue). The images are representative of three mice, with ~5 fields of view per sample. Scale bars, 100 μm.

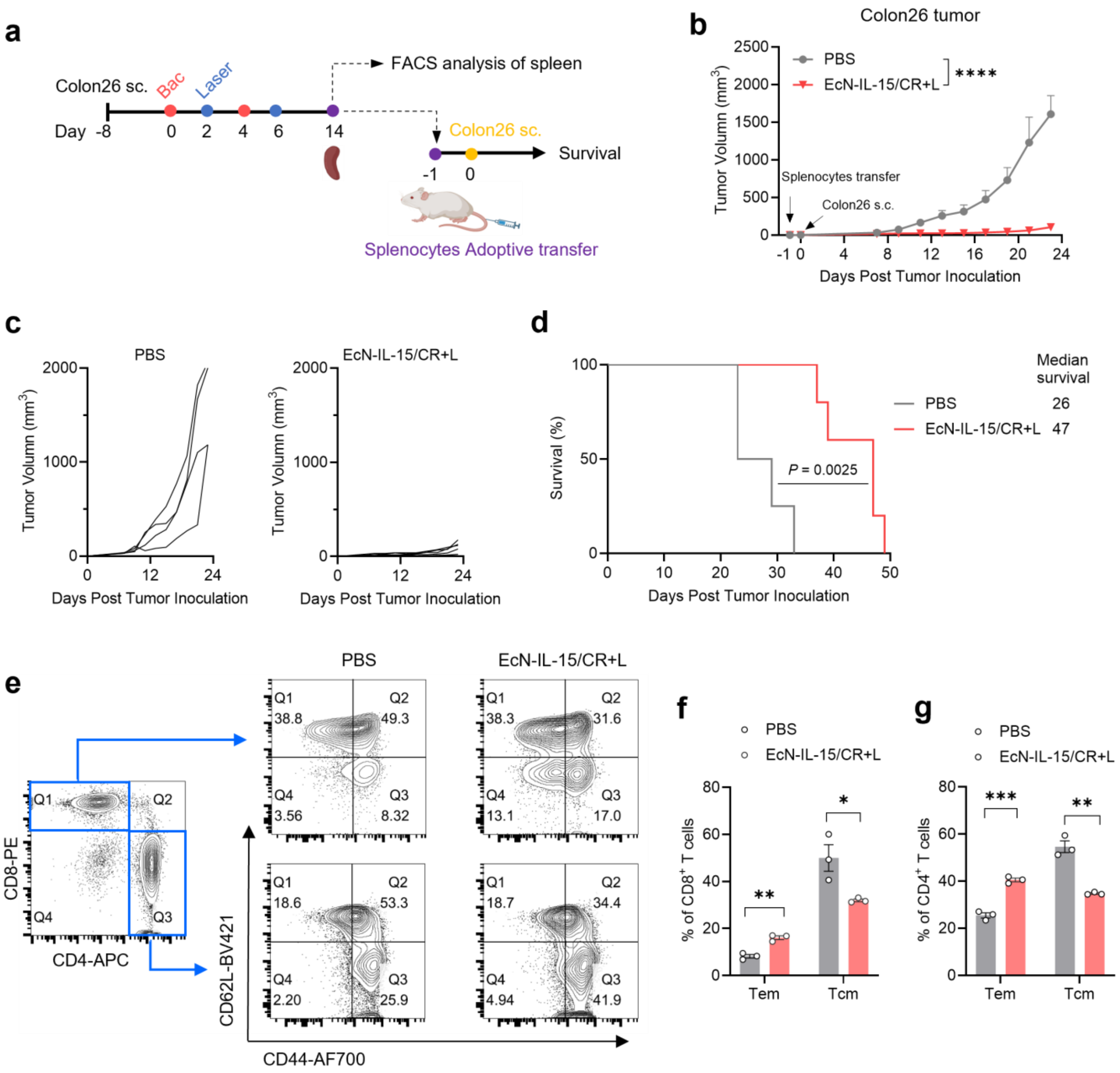

Figure S17. Photothermal EcN-IL-15/CR promotes memory T-cell generation. Related to Figure 5.

(**a**) Schematic illustration of the adoptive splenocytes transfer experiment.

(**b-d**) Tumor growth and survival analysis. ~1×10^7 splenocytes were transferred into naïve mice, followed by Colon26 tumor challenge; n = 4–5 biological replicates.

(**e-g**) Flow cytometric analysis of memory T-cell subsets in the spleens. Effector memory T cells (Tem, $CD62L^{lo}CD44^{hi}$) and central memory T cells (Tcm, $CD62L^{hi}CD44^{hi}$) were quantified from $CD8^+$ (**f**) and $CD4^+$ (**g**) T cells; n = 3 biological replicates.

Data are presented as means ± SEM. *P*-values were analyzed by two-tailed unpaired Student's t-test (**f**, **g**), two-way ANOVA with Sidak's multiple comparisons (**b**), or Mantel–Cox log-rank test (**d**). **P* < 0.05, ***P* < 0.01, ****P* < 0.001, *****P* < 0.0001.

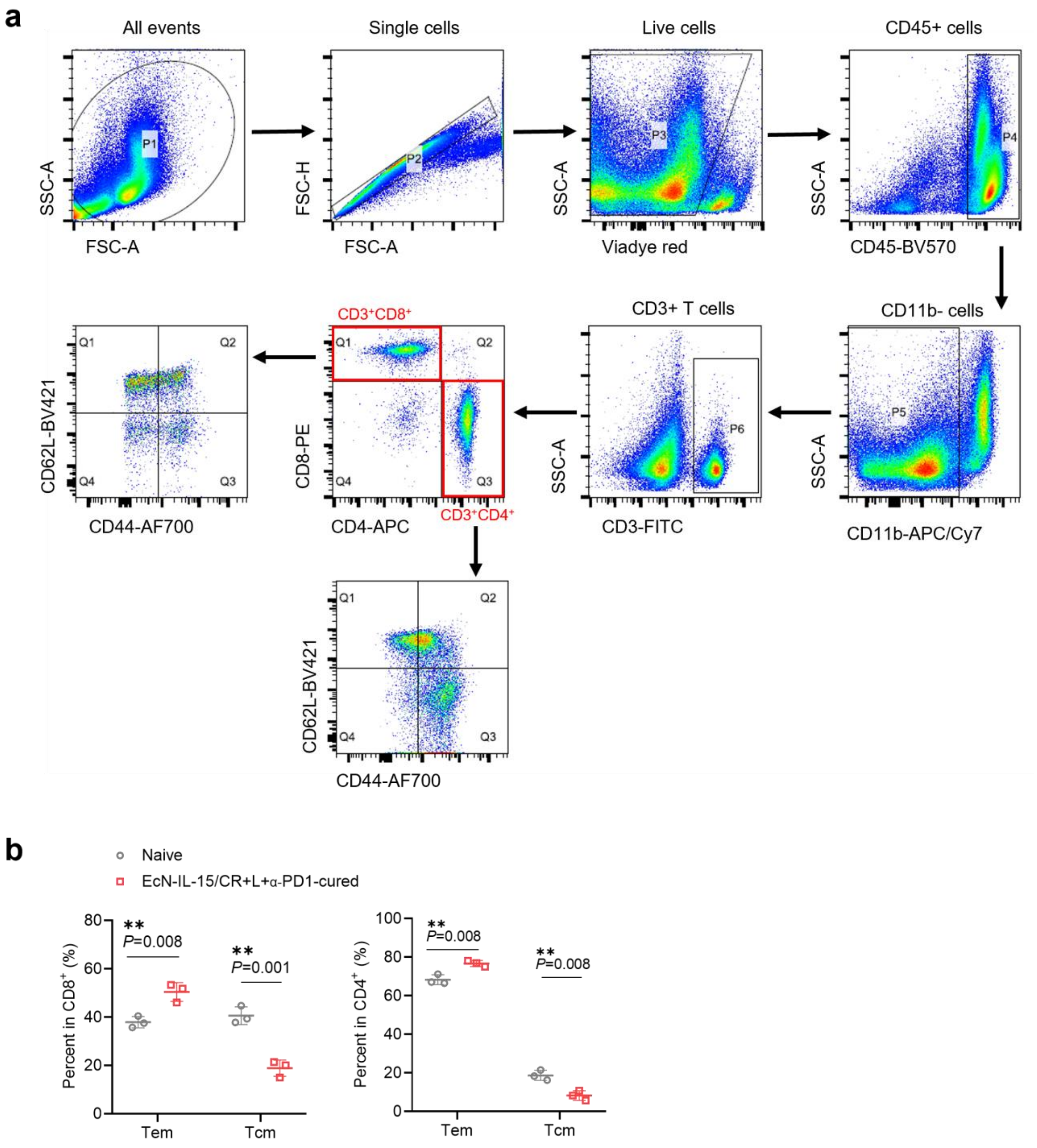

Figure S18. Analysis of memory T cells in spleens. Related to Figures 6.

(a) Gating strategy for flow cytometric analysis of memory T cells in spleens.

(b) Flow cytometric analysis and quantification of memory T cells in spleens after rechallenging. Flow cytometric analysis and quantification of Tem and Tcm subsets from $CD8^+$ and $CD4^+$ T cells in the spleens of the rechallenged mice; n = 3 biological replicates.

Data are presented as means ± SEM. *P* values were calculated by two-tailed unpaired Student's t-test. $^{**}P < 0.01$.

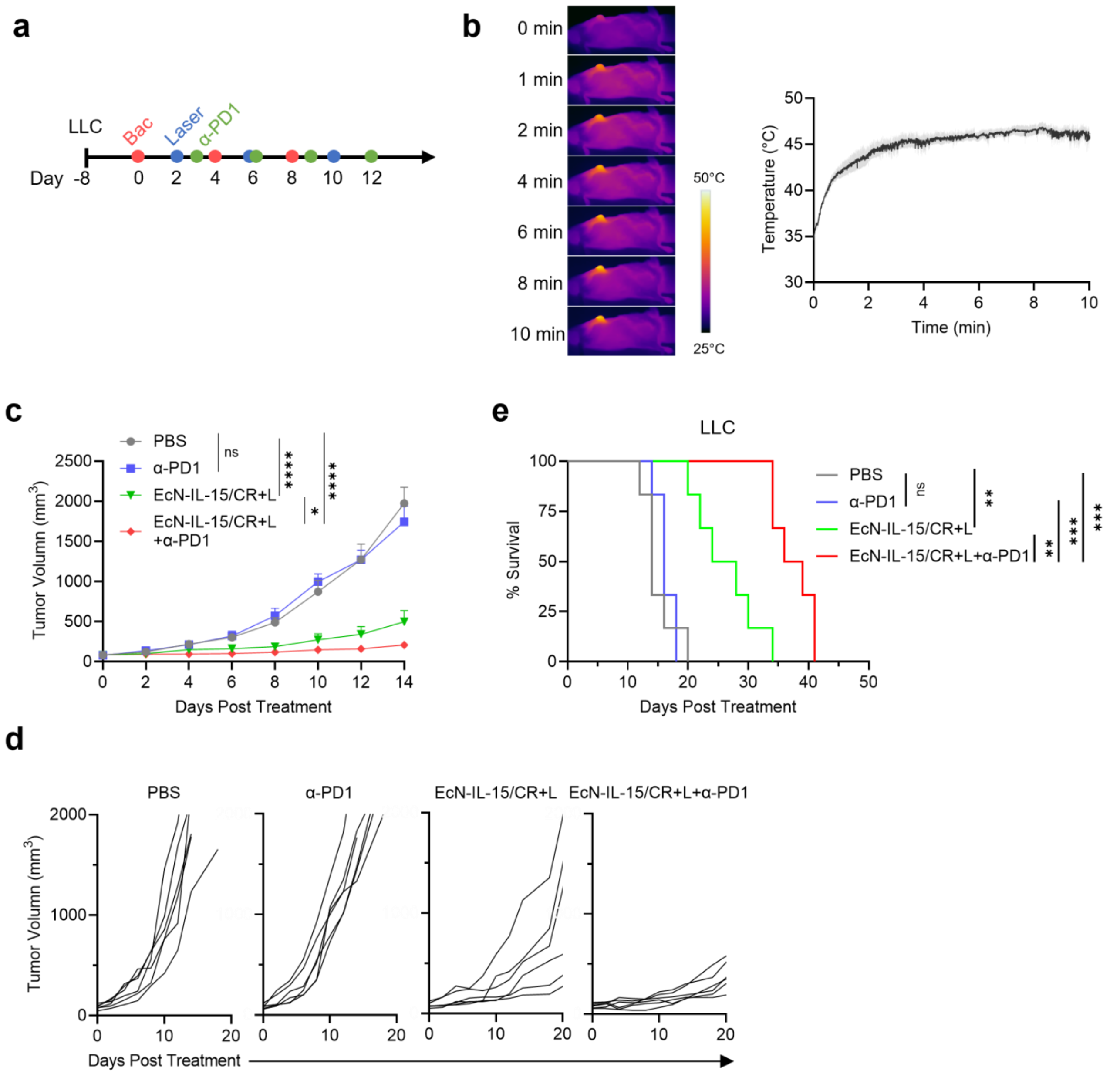

Figure S19. Photothermal EcN-IL-15/CR synergizes with PD-1 blockade to control immune-cold tumors. Related to Figure 6.

(**a**) Treatment schedule of EcN-IL-15/CR+L with anti-PD1 combination therapy in the subcutaneous LLC tumor. LLC-bearing mice were intravenously injected with EcN-IL-15/CR (8×10^7 CFU), followed by 808 nm laser irradiation 48 hours post-injection (3 injections in total). Mice were intraperitoneally injected with anti-PD1 antibody (250 µg) every 3 days (4 injections in total) as indicated; n = 6 biological replicates.

(**b**) Representative thermal images and temperature variations of LLC tumor-bearing mice exposed to 808 nm laser irradiation (0.9 W cm^{-2}, 10 min) after intravenous injection of EcN-IL-15/CR; n = 3 biological replicates.

(**c-d**) LLC tumor growth curves; n = 6 biological replicates.

(**e**) Kaplan–Meier survival curves for LLC tumor-bearing mice; n = 6 biological replicates.

Data are presented as means ± SEM. *P*-values were analyzed by two-way ANOVA with Holm-Sidak's multiple comparisons

test (**c**), or Mantel–Cox log-rank test (**e**). *$P < 0.05$, **$P < 0.01$, ***$P < 0.001$, ****$P < 0.0001$; ns, not significant.

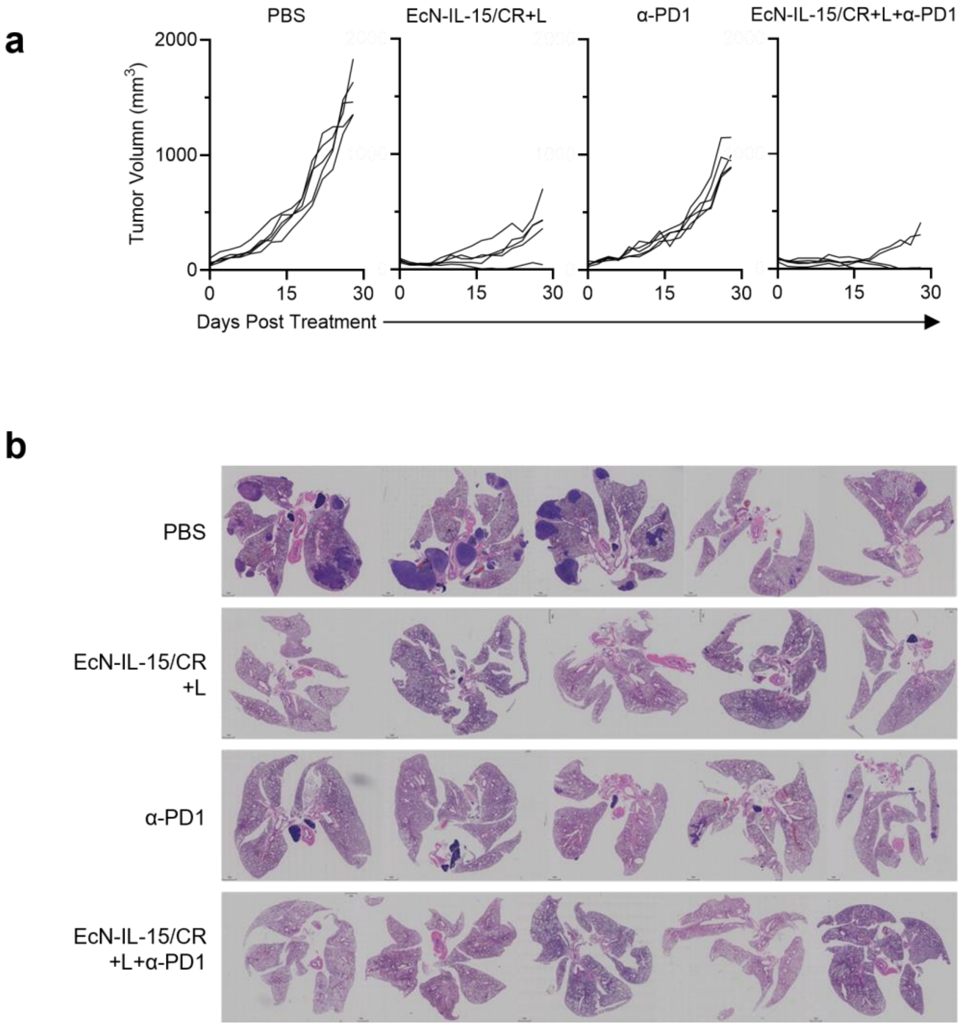

Figure S20. Photothermal EcN-IL-15/CR synergizes with PD-1 blockade to inhibit tumor metastasis in an orthotopic breast cancer model. Related to Figure 7.

(**a**) Tumor growth curves of individual mice, corresponding to Figure 8d. n = 5 biological replicates.

(**b**) H&E-stained lung slices from different groups of 4T1-bearing mice. The lungs of mice treated with EcN-IL-15/CR+L in combination with anti-PD1 therapy showed little metastasis; n = 5 biological replicates.

Table S1. Coding sequence of IL-15. Related to STAR Method.

The original sequence of pNeae2-IL-15<br>HPVGLLARVPLSLYSGHPVGLLARVPLSLYSGLSGRSDNHGGGSGGGSGIHVFILGCVSVGLPKTEANWIDVRYDL<br>EKIESLIQSIHIDTTLYTDSDFHPSCKVTAMNCFLLELQVILHEYSNMTLNETVRNVLYLANSTLSSNKNVAESGCK<br>ECEELEEKTFTEFLQSFIRIVQMFINTS
The Optimized (*for Escherichia coli*) sequence of pNeae2-IL-15<br>CATCCGGTGGGCCTGCTGGCGCGCGTTCCGTTAAGCCTGTATAGTGGCCATCCGGTTGGCTTACTGGCGCGCG<br>TGCCGCTGAGCCTGTATAGCGGCCTGAGCGGTCGCAGCGATAACCATGGTGGCGGCAGCGGCGGCGGTAGTG<br>GCATTCATGTGTTTATTCTGGGCTGCGTGAGCGTGGGCCTGCCGAAAACCGAAGCGAACTGGATTGATGTGCG<br>CTATGATCTGGAAAAAATTGAAAGCCTGATTCAGAGCATTCATATTGATACCACCCTGTATACCGATAGCGATTT<br>TCATCCGAGCTGCAAAGTGACCGCGATGAACTGCTTTCTGCTGGAACTGCAAGTGATTCTGCATGAATATAGC<br>AACATGACCCTGAACGAAACCGTGCGCAACGTGCTGTATCTGGCGAACAGCACCCTGAGCAGCAACAAAAA<br>CGTGGCGGAAAGCGGCTGCAAAGAATGCGAAGAACTGGAAGAAAAAACCTTTACCGAATTTCTGCAGAGCT<br>TTATTCGCATTGTGCAGATGTTTATTAACACGAGC
